# What Doesn't Kill You Makes You Stronger? Examining Relationships Between Early‐Life Stress, Later‐Life Inflammation and Mortality Risk in Skeletal Remains

**DOI:** 10.1002/ajpa.70005

**Published:** 2025-02-05

**Authors:** B. R. Wigley, E. C. Stillman, E. Craig‐Atkins

**Affiliations:** ^1^ School of Biosciences University of Sheffield Sheffield UK; ^2^ School of Mathematical and Physical Sciences University of Sheffield Sheffield UK; ^3^ School of History, Philosophy and Digital Humanities University of Sheffield Sheffield UK

**Keywords:** developmental plasticity, fluctuating asymmetry, geometric morphometric methods, inflammation, life‐course bioarcheology, thrifty phenotype

## Abstract

**Objectives:**

This paper explores conflicting perspectives on the adaptive significance of phenotypic plasticity during fetal and early postnatal development and the impact that stressors experienced during this critical early‐life period have on later‐life morbidity and mortality risk.

**Methods:**

The sample (*n* = 216) comprised archeologically‐recovered human skeletons. A geometric morphometric (GM) method was employed to evaluate first permanent molar (M1) fluctuating asymmetry (FA) and provide a proxy for early‐life stress. Shifts in later‐life physiology were inferred through two inflammatory lesions: periosteal new bone formation (PNBF) and periodontal disease (PD). To explore mortality risk, age‐at‐death was estimated through dental development for skeletally immature individuals (*n* = 104) and through senescent skeletal changes for mature skeletons (*n* = 112).

**Results:**

Significant differences were found in M1 FA between groups, with the immature cohort associated with elevated FA. Within‐group analysis revealed age‐at‐death in the immature group had a significant positive relationship with M1 FA and PD presence. In the mature group, alongside sex and the co‐occurrence of PD and PNBF, FA was a significant predictor of a shorter life. Higher FA was also associated with active and bilaterally expressed PNBF.

**Conclusions:**

It is theorized that early‐life stress, if survived, programmed a hyperinflammatory response to environmentally‐mediated physiological perturbations which increased the chances of survival during subsequent development but also elevated later‐life mortality risk. Findings demonstrate a complicated relationship between developmental stress and physiological shifts that helps to illustrate the adaptive significance of early‐life programming and support the Thrifty Phenotype hypothesis.

## 1 Introduction

Phenotypic traits are observable characteristics which result from the interaction of genes with environmental influences. During sensitive developmental windows, which are periods of elevated somatic plasticity, traits are malleable and stressors can provoke phenotypic alterations that persist throughout life (Gowland and Caldwell 2023, 520). Thus, while stressors endured over life accumulate and coalesce to influence development, morbidity and mortality risk, those experienced during fetal life and the first postnatal year (a period referenced as “early life” hereafter) exercise a disproportionate impact (Agarwal 2016; Barker 2012; Gowland and Caldwell 2023). It is, however, debated whether exposure to stressors during development is associated with adaptations that increase survivorship and reproductive success (e.g., Bateson, Gluckman, and Hanson 2014) or, alternatively, if adaptations that promote survival initially are connected to trade‐offs that diminish later resilience, increasing morbidity and mortality risk (e.g., Barker and Osmond 1986; Hales and Barker 2013; Wells 2010). Put another way, is there any truth in the popular aphorism “what doesn't kill you makes you stronger”? To answer this question, stress‐related variation in mortality risk and later‐life inflammation are explored with reference to these conflicting hypotheses and through the examination of an archeologically‐recovered skeletal sample (*n* = 216).

Due to the timing of their development, first permanent molar (M1) crowns were assessed as a proxy for early‐life stress. M1 morphogenesis is controlled by signaling centers located at cusps, begins at approximately sixteen weeks in utero, and continues throughout the fetal period (Antoine and Hillson 2016, 223–224; Jernvall and Jung 2000; Kenessey, Stojanowski, and Paul 2024, 2; Scott and Turner 1997, 76). Although enamel formation towards the cervical region is not complete until about the third postnatal year, M1 cusps begin to mineralize around birth with cuspal enamel formation complete towards the end of the first postnatal year or soon thereafter (Reid and Dean 2006, 334). Mineralization of the signaling centers renders them inert. Thus, the spatial patterning of M1 coronal features is determined and fixed during the early‐life period (Jernvall and Jung 2000; Kenessey, Stojanowski, and Paul 2024, 2). A geometric morphometric (GM) approach was used to assess M1 coronal morphology. Specifically, Procrustean techniques isolated shape variation so that M1 fluctuating asymmetry (FA) could be evaluated and used as a proxy for early‐life stress (Wigley, Stillman, and Craig‐Atkins 2024). Inflammation in later‐life (i.e., beyond the early‐life period), was inferred through two skeletal stress markers: periosteal new bone formation (PNBF) and periodontal disease (PD) (Ogden 2008; Weston 2008). Age‐at‐death was estimated with reference to developmental as well as senescent changes (AlQahtani, Hector, and Liversidge 2010; Boldsen et al. 2002). It was therefore possible to model links between developmental experience, later physiological outcomes, and mortality risk.

## 2 Background

### 2.1 Stress and Critical Periods

A stressor can be defined as any stimulus that disrupts homeostasis and provokes a physiological counter‐response, such as a trade‐off in resource allocation. Due to the plethora of stimuli that can cause such disruptions, stress is generally regarded as non‐specific in nature (Escós et al. 2000, 331; Selye 1973). Early‐life (i.e., the fetal period to the end of the first postnatal year) has been shown to be especially stress‐sensitive and, due to elevated phenotypic plasticity, critical in influencing growth, development, morbidity and survivorship (Agarwal 2016; Barker 2012; Gowland and Caldwell 2023; McPherson 2021).

The life‐course outcomes of Dutch Famine survivors illustrate well the importance of early life experience. During the winter of 1944–1945, daily nutritional intake often fell below 1000 cal in the occupied Netherlands, leading to widespread malnutrition. Follow‐up studies over subsequent decades found that maternally dependent offspring who experienced the famine during early life were subject to higher rates of chronic diseases, infections, and mortality risk in later life than their peers who escaped exposure to nutritional shortages during this crucial developmental period (Lopuhaa et al. 2000; Ravelli et al. 1998; Roseboom et al. 2000; Roseboom, van der Meulen, and Ravelli 2001). These impacts were particularly pronounced when fetuses were affected in the third trimester of development (Bleker et al. 2021; Roseboom, van der Meulen, and Ravelli 2001, 95).

Research has highlighted that environmental factors and their impact on maternal nutrition and health are likely a key stressor during critical periods of development and articulate that early‐life adversity can be robustly associated with life‐long increases in morbidity and mortality risk. Consequently, they have contributed to fundamental shifts in the conceptualization of health, including the formulation of the Developmental Origins of Health and Disease Hypothesis (Agarwal 2016; Barker 2012; Gluckman, Hanson, and Beedle 2007; Gluckman, Hanson, and Buklijas 2010; Gowland and Caldwell 2023).

### 2.2 Adaptive Significance of Developmental Plasticity

Early work proposed that alterations to phenotype in response to early‐life cues were often deleterious to health. The Thrifty Phenotype hypothesis developed by Hales and Barker (1992, 2001, 2013) is an example. This hypothesis took inspiration from what Neel (1962) referred to as a “thrifty” genotype, or a genetically programmed metabolism which was overly‐efficient at processing nutritional resources. While beneficial when access to food is irregular or unreliable, in relatively stable and plentiful environments, this leads to poor metabolic regulation. Building on this, Hales and Barker (1992, 2001, 2013) proposed that for individuals experiencing poor nutrition during early life, there is a reduction in investment in the production of cells key to normal metabolic functioning. This thrift, or constraint, during development is initially adaptive as it prevents scarce resources being deployed unnecessarily, but can lead to pathologically impaired functioning (e.g., increased susceptibility to Type II diabetes) if exposed to less adverse environmental conditions later in life (Hales and Barker 2013, 1219). Thrifty phenotypes are thus ones that promote survival during developmental adversity, but increase later‐life morbidity and mortality risk. Although mechanisms have been debated, the Thrifty Phenotype hypothesis has been employed to explain differentials in frailty (i.e., age‐adjusted vulnerability) that have contributed to *inter alia* increased prevalence of degenerative disease, metabolic dysfunction, and growth deficits (e.g., Pomeroy et al. 2012; Weisensee 2013; Vaupel 1988, 277).

Not all researchers have viewed adaptations to early‐life stress through such a negative lens, however. The Predictive Adaptive Response (PAR) hypothesis presents an alternative perspective on the significance of phenotypic plasticity (Bateson et al. 2004; Gluckman, Hanson, and Beedle 2007; Gluckman, Hanson, and Buklijas 2010; Wells 2007, 331). Although the PAR hypothesis does not rule out the capacity for phenotypic variation to provide immediate benefits at the expense of later‐life trade‐offs, predictive responses are characterized as alterations following developmental cues which increase fitness during reproductive years. PARs maximize the chances of having offspring and therefore promote long‐term organismal success (Bateson, Gluckman, and Hanson 2014, 2358; Gluckman, Hanson, and Beedle 2007, 4; Gluckman, Hanson, and Buklijas 2010, 9; Lu et al. 2019, 252; Wells 2007, 335). To illustrate, a review by Kuzawa (2007) found sexually dimorphic features (e.g., stature and hormone expression) were positively associated with birth weight, a widely‐used proxy for fetal stress and experience of in utero adversity. Kuzawa (2007) proposed that investment in somatically costly dimorphic traits had been calibrated in relation to early‐life nutritional cues to increase the likelihood of long‐term survival and reproductive fitness.

### 2.3 Exploring Relationships Between Early‐Life Stress and Later‐Life Outcomes in Skeletal Assemblages

Later‐life morbidity and mortality risk have been associated with early‐life stress (e.g., Barker and Osmond 1986). Various theories have attempted to explain the adaptive significance of stress‐induced phenotypic alterations (e.g., Bateson, Gluckman, and Hanson 2014; Hales and Barker 2013). One of the main differences between these, and the question addressed in this paper, is the extent to which adaptations result in short‐term or long‐term benefit.

Addressing this question in archeological skeletal remains requires careful consideration of how stress is evaluated. This is especially the case for developmental stress as the remains of individuals who are still growing represent a biased cohort of “non‐survivors”, while the traces of developmental experience can be difficult to parse from the impact of perturbations experienced in later‐life for skeletally mature individuals (DeWitte and Stojanowski 2015, 416–418; Wood et al. 1992, 349). The assessment of random deviations to perfect symmetry (i.e., fluctuating asymmetry) reflects developmental instability and can be used to infer stress experience (Klingenberg 2015; Graham et al. 2010; Van 1961). By focusing on teeth, which form at specific times and do not remodel throughout life, critical periods can be evaluated in the remains of both adults and non‐adults (Wigley, Stillman, and Craig‐Atkins 2024, 5). For example, Moes, Kuzawa, and Edgar (2024) evaluated FA in the deciduous dentition to explore the impact of gestational temperature on development. It was found that teeth forming during the second and third months in utero exhibited significantly higher dental FA. From this, it was suggested that fetal development is particularly sensitive to environmental conditions during the first trimester. Elevated FA and dental stress markers have also been associated with morbidity risk (e.g., Dewitte and Wood 2008; O'Donnell and Moes 2020). Weisensee (2013), for instance, found significantly higher FA in individuals known to have died from degenerative conditions, linking increased developmental instability with elevated vulnerability to somatic dysregulation in later‐life.

One way of evaluating later‐life phenotype and its relation to morbidity in skeletal remains is through the examination of skeletal stress markers such as periodontal disease (PD) and periosteal new bone formation (PNBF). Investigating phenotype from these lesions is not uncomplicated, however, as they have been linked to different risk factors. As it largely results from the gradual accumulation of bacterial plaque, PD has been associated with diet, oral hygiene, and age‐related degeneration (Caruso and Nikita 2024, 13; Larsen 1997, 77; Ogden 2008). In contrast, PNBF has often been connected to infection or trauma (Roberts and Buikstra 2019; Roberts 2019; Weston 2008, 49). However, both lesions frequently share an inflammatory pathophysiology (Caruso and Nikita 2024, 13; DeWitte and Bekvalac 2011, 615; Weston 2008, 49). As such, it has been proposed that PD and PNBF can be employed to reconstruct inflammatory phenotype (Crespo 2021, 79–80). This assertion is supported by findings from living populations. Although usually associated with age‐related degeneration, PD has been observed in children alongside increased morbidity in inflammatory disorders (Chipirliu, Craciun, and Matei 2024; Koutsochristou et al. 2015).

The assessment of inflammatory phenotype is important as inflammation is a protective response of the innate immune system involving immune cells, signaling proteins, and the vascular system. An effective inflammatory reaction eliminates the cause of cellular injury and initiates repair and removal of damaged tissues (Crespo 2021, 76–79; Oishi and Manabe 2018). Inflammatory phenotype is therefore key to long‐term morbidity and mortality risk.

Connections between developmental stress, later‐life physiology, and variation in mortality risk were explored by assessing M1 FA, PNBF and PD, as well as estimates of age‐at‐death. The use of skeletal proxies for stress is a complementary approach to the early work, which largely focused on links between developmental nutrition and metabolic morbidity in modern populations with longitudinal medical records (e.g., Hales and Barker 1992). Later research has successfully employed the Thrifty Phenotype hypothesis to explained diverse aspects of skeletal variation (e.g., Pomeroy et al. 2012; Wells, DeSilva, and Stock 2012) and it is increasingly being acknowledged that the application of such hypothetical frameworks to bioarchaeological investigations can generate meaningful insights (e.g., Agarwal 2016, 131–133). To the authors' knowledge, the use of this suite of stress markers to explore the adaptive significance of phenotypic plasticity is novel. The use of M1 FA allows stress experience at a key period of life to be captured, while inflammatory lesion prevalence and age‐at‐death estimates can be employed to reconstruct morbidity (and potentially the extent of immune competence/incompetence) and mortality risk. These stress markers provide an opportunity for hypotheses to be tested in past populations. Necessarily, inferences must be made with caution due to the lack of in vivo data and limitations associated with sampling archeological populations (DeWitte and Stojanowski 2015, 399; Wood et al. 1992). Two hypotheses were proposed to test the Thrifty Phenotype and PAR hypotheses.

Hypothesis 1 *If the Thrifty Phenotype hypothesis were to hold true, it was predicted that M1 FA, as a proxy for early‐life stress, would be linked to evidence that implied initially adaptive shifts in phenotype, but that ultimately individuals with elevated FA would have higher frequencies of stress markers (PNBF and PD) and/or earlier age‐at‐death*.

Hypothesis 2 *Following the PAR framework, higher M1 FA should be linked to a lower frequency of skeletal stress markers and higher age‐at‐death estimates. This would suggest elevated stress had promoted resilience throughout life, reducing later‐life morbidity and mortality risk*.

## 3 Materials

The skeletal remains of 216 individuals curated by the Archeology and Heritage Science Facility at the University of Sheffield were assessed. The remains originate from four assemblages of medieval and post‐medieval date: the Black Gate cemetery, Newcastle (BG); St Hilda's Church, South Shields (SS); St Lawrence's Church, Warwick (WS); and All Saints' Church, York Barbican (YB) (Table 1). Using data from multiple sites expanded sample size, enhancing statistical power. The populations represented by these assemblages experienced variation in living conditions and stress exposure.

**TABLE 1:** Summary of the skeletal sample, highlighting variations in date as well as social, cultural, and economic context.

Site	Sample size (immature/mature)	Date	Characteristics
Black Gate, Newcastle‐upon‐Tyne (BG)	84 (37/47)	7th–11th century CE	Dispersed rural community Mixed social status
St Hilda's, South Shields (SS)	33 (14/19)	18th–19th century CE	Industrialized port town Highly polluted
St Lawrence's, Warwick (WS)	29 (20/9)	11th–15th century CE	Rural population Documented periods of resource scarcity
All Saints', York Barbican (YB)	70 (33/37)	11th–16th century CE	Densely‐populated urban center Prosperous commercial hub

The Black Gate collection dates to the early‐medieval period and is associated with a community comprising both secular and religious elites along with a dispersed rural population likely exposed to seasonal variations in resource availability (Mahoney Swales 2019; Nolan, Harbottle, and Vaughan 2010). The remains interred at St Lawrence's Church on the outskirts of Warwick were likewise a medieval rural population, but from a later date. The community faced repeated famines and bouts of plague (i.e., Black Death) which affected much of Britain during the 14th century and were likely exacerbated in the Warwick region due to the over‐exploitation of the surrounding landscape (Gethin n.d.; Harley 1958, 18; John 1997; Slavin 2013). The York Barbican and Warwick collections roughly correspond in date and therefore experienced the same periods of nationwide resource deprivation and epidemics. It is likely, however, that due to its position as a commercial hub the population of York had the capacity to mitigate some of the most severe effects of nutritional shortages through trade and exchange (Barrett, Locker, and Roberts 2004; Bruce 2003; Goldberg 2019; McIntyre and Bruce 2010; Tilliot 1961). The individuals from South Shields are associated with a town experiencing rapid industrialization where residents, most of whom were of lower socioeconomic status, were exposed to arduous labor practices and an ever‐increasing range of environmental pollutants (Raynor, McCarthy, and Clough 2011; Report of the Commissioners 1845).

## 4 Methods

### 4.1 Geometric Morphometric Methods: M1 Fluctuating Asymmetry

A geometric morphometric evaluation of dental fluctuating asymmetry was conducted to explore developmental stress. GM methods were selected as they represent a highly sensitive analytical toolkit capable of detecting the small, random departures from perfect symmetry which characterize FA (Graham et al. 2010; Klingenberg 2015). This evaluation focused on M1 crowns. M1s form through a stress‐sensitive process which begins in utero and determines the spatial patterning of the crown's occlusal features (Scott and Turner 1997, 76; Jernvall and Jung 2000). Subsequent enamel mineralization, which begins at the signaling centers responsible for morphogenesis, largely fixes the shape of M1 occlusal features towards the end of the first postnatal year (Jernvall and Jung 2000; Kenessey, Stojanowski, and Paul 2024, 2; Reid and Dean 2006, 334). By assessing the M1 occlusal surface it was possible to employ FA as a proxy for stress experienced during the early‐life period (Wigley, Stillman, and Craig‐Atkins 2024).

From the 216 individuals sampled, 154 maxillary first permanent molar (M^1^) and 147 mandibular first permanent molar (M_1_) antimeric pairs were assessed. A Canon EOS 250D DSLR camera attached to a Kaiser Copy Stand and fitted with an AET‐CS Auto Extension Tube was used to image teeth. M1s were placed so that the cemento‐enamel junction was parallel to the camera lens when photographed. The location of homologous landmarks (Table 2) was defined through *x–y* coordinates digitized on each image using the R package *Stereomorph* (Olsen 2015; Olsen and Westneat 2015). Similarly, a curve was drawn and subsampled by 20 equidistant semi‐landmarks to describe the occlusal outline of each tooth. As FA is exceptionally sensitive to error, three replicate measures were taken per tooth by the first author so that intra‐observer error could be evaluated (Palmer 1994; Wigley, Stillman, and Craig‐Atkins 2024, 6–7).

**TABLE 2:** Maxillary and mandibular first permanent molar landmarks.

Tooth	Landmark no.	Description
Maxillary M1	1	The center of the mesial fovea, at the most mesial extension of the sagittal fissure
	2	The intersection of the sagittal fissure by the buccal fissure
	3	The intersection of the sagittal fissure by the lingual fissure
	4	The center of the distal fovea located at the most distal extension of the sagittal fissure
	5	Paracone apex
	6	Metacone apex
	7	Protocone apex
	8	Hypocone apex
Mandibular M1	1	The center of the mesial fovea, at the most mesial extension of the longitudinal fissure
	2	The intersection of the longitudinal fissure by the mesiobuccal fissure
	3	The intersection of the longitudinal fissure by the lingual fissure
	4	The intersection of the longitudinal fissure by the distobuccal fissure
	5	The distal fovea located at the most distal extension of the longitudinal fissure and, when present, its intersection with the buccal and lingual foveal fissures
	6	Protoconid apex
	7	Hypoconid apex
	8	Metaconid apex
	9	Entoconid apex
	10	Hypoconulid apex

With M1s represented through configurations of coordinate points it was possible to isolate shape variation. This included ensuring a point‐to‐point correspondence between semi‐landmarks by sliding each one along a line tangent to the chord connecting adjacent outline points to reduce bending energy (Dryden and Mardia 2016, 368). To isolate shape variation, a Generalized Procrustes Analysis (GPA) was employed to relocate, rescale, and rotate configurations to minimize the sum of squared distances between configurations and register them in Kendall's shape space. To circumvent the non‐Euclidean geometry of Kendall's shape space, GPA‐aligned configurations were projected into tangent linear space where the sum of squared differences between them could be explored through standard statistical techniques (Bookstein 1991; Dryden and Mardia 2016).

A two‐way, mixed model Procrustes Analysis of Variance (ANOVA) decomposed sample‐level variation. The interaction term of the Procrustes ANOVA quantified random deviations between sides without a directional bias (i.e., fluctuating asymmetry) and the error term measured the contribution of between‐replicate differences (i.e., intra‐observer error). An *F* value was derived from the Procrustes ANOVA procedure by dividing the mean square for the interaction term by that of the error term (Palmer 1994; Klingenberg and McIntyre 1998). This provided a ratio of FA to error, contrasting random, stress‐induced variation to the impact of error in the data collection process (Collyer and Adams 2024, 188–191). FA was deemed significant when the observed *F* value exceeded the 95th percentile (i.e., *p* < 0.05) of an empirical sampling distribution of random pseudo values generated from the residuals of null linear models added to the fitted values (Collyer and Adams 2018; Collyer and Adams 2024, 185). These procedures were implemented using the R package *Geomorph* (Adams et al. 2023).

Contingent upon significant results, an individual measure of fluctuating asymmetry was calculated. First, M1 replicates were averaged to mitigate the impact of observer error. Average left and right configurations associated with an individual were then subtracted from one another to give a coordinate configuration reflecting asymmetric differences between antimeric teeth. To adjust for directional biases in the overall skeletal sample, the difference between the sample's average left and right shapes was then subtracted. The values in the resulting configuration were squared, summed over *x* and *y* coordinates, and the square root found to give an individual estimate of antimeric FA (Klingenberg and McIntyre 1998, 1375; Bookstein 1991, 269; Wigley, Stillman, and Craig‐Atkins 2024, 11–12). To provide a more robust measure of developmental stress, isomeric FA scores were averaged to produce a single composite FA score that approximately represented an individual's early‐life stress experience (Graham and Ozener 2016; Wigley, Stillman, and Craig‐Atkins 2024). By calculating an individual estimate, it was possible to explore between and within group patterns (i.e., analyzes were not limited to comparisons of mean values and FA scores could, for instance, be regressed on age estimates).

### 4.2 Osteological Methods: Demographic and Paleopathological Variables

To generate demographic data, initially individuals were separated into skeletally “immature” and “mature” cohorts. Skeletal immaturity was identified here through the presence of teeth that were not fully developed and erupted. For these immature individuals, dental formation and eruption was assessed using the atlas of AlQahtani, Hector, and Liversidge (2010) to provide an estimate of age‐at‐death. Skeletally mature individuals had fully formed and erupted dentition. For mature skeletons, age was estimated with reference to late‐fusing epiphyses (i.e., the medial clavicle, spheno‐occipital synchondrosis, vertebral epiphyseal rings, and sacrum) and stages of senescent change in the pubic symphysis and auricular surface. These were graded after Milner et al. (2019). The ADBOU transition analysis (TA^3^ version 0.8.0) was employed to evaluate these observations and produce a Maximum Likelihood point estimate of age with reference to stages of change in a known age‐at‐death sample (Getz 2020; Boldsen et al. 2002).

Sex was estimated for mature skeletons through the assessment of dimorphic features in the pelvis and skull. Morphological characteristics were scored along a 5‐grade scale which was then simplified (i.e., grades 1–2 combined into a “female” group, 4–5 into a “male”, while sex was considered indeterminate for remains with a grade of 3). An individual's average score was used to place them in a particular group (White and Folkens 2005, 385–397; Buikstra and Ubelaker 1994, 15–20; Ferembach, Schwidetzky, and Stloukal 1980).

Physiology and morbidity in later‐life was explored through PNBF and PD presence. PNBF was recorded as present when plaques of elevated bone could be identified on the surfaces of larger appendicular long bones (i.e., femora, tibiae, fibulae, humeri, radii and ulnae) (Weston 2008, 51). When dealing with younger individuals, PNBF was graded present only when circumscribed plaques were distinct from the underlying cortical bone, to prevent confusion with normal bone growth (Lewis 2018, 114–116). To maximize sample size and increase statistical power, incomplete skeletons were included in the study. For these individuals, PNBF was recorded absent when no periosteal plaques could be discerned on the available long bones (i.e., for PNBF to be recorded as present/absent, a minimum of one well‐preserved long bone had to be observable). Smaller skeletal elements, which do not survive as well in archeological contexts were not assessed to expedite data collection. “Remodeled” and “active” lesions were differentiated. That is, plaques of lamellar bone with either a well‐organized and striated or dense, sclerotic appearance were considered indicative of a resolved or remodeled/inactive inflammatory response, while a lesion with a woven, porous or disorganized appearance was interpreted as evidence of a process still active at time‐of‐death (Weston 2008, 51–52). Where possible, it was noted if periosteal lesions had a unilateral or bilateral distribution, with the latter being interpreted as more likely associated with systemic inflammation rather than a localized response to trauma (Weston 2012). PD was diagnosed and recorded after the criteria established in Ogden (2008, 293) (Table 3).

**TABLE 3:** Grading of PD after Ogden (2008, 293).

Grade	Description of alveolar crest	Diagnosis
x	No observation possible	Not applicable
0	Alveolar bone meets tooth at a knife‐edged acute angle	No disease
1	Alveolar margin is blunt, flat‐topped with a slight rim	Mild PD
2	Rounded and porous margin, with a trough 2–4 mm deep	Moderate PD
3	Ragged and porous with an irregular trough > 4 mm deep	Severe PD

To evaluate PNBF and PD frequency, prevalence rates were calculated. Crude prevalence rate (CPR) was found as the percentage of individual skeletons affected for whom an observation was possible. True prevalence rate (TPR) was computed as the percentage of bones (or, for PD, regions of the alveolus surrounding molars) exhibiting pathological changes where observations were possible (Waldron 1994).

### 4.3 Statistical Analysis: Predicting Life‐Course Outcomes

A suite of standard statistical techniques was employed to explore relationships between factors. To be amenable to these procedures and mitigate against the GPA's arbitrary scaling, composite FA scores were log transformed and scaled so that they were centered about a mean of zero with a standard deviation of ±1 (Grus 2015; Wigley, Stillman, and Craig‐Atkins 2024, 15). Similarly, as age estimates were positively skewed, they were log transformed.

Regression procedures were used to explore the relationships between stress markers and demographic variables so that the hypotheses relating to the Thrifty Phenotype and PAR models could be tested. Linear regression was employed when the response variable was quantitative (i.e., FA scores and age estimates) and binary logistic regression was employed when modeling the presence/absence of pathological lesions. Regression models were constructed with a stepwise selection process in which independent variables were iteratively added and subtracted to find the best combination of explanatory factors (Gelman and Hill 2007). Given differences in prevalence rates noted in exploratory data analysis (below), for the purposes of modeling PNBF presence, later‐medieval individuals from York Barbican and Warwick were combined into one group, while those from Black Gate and South Shields formed another. Similarly, when exploring which factors predicted PD presence, “absent” and “mild” cases were combined while “moderate” and “severe” lesions were grouped (Ogden 2008, 293); medieval sites were contrasted with post‐medieval South Shields. The proportion of variation explained by models was measured through the coefficient of determination (*R* ^2^); an adjusted coefficient was employed (adj. *R* ^2^) for linear models and McFadden's pseudo‐*R* ^2^ was used for logistic regression (Hardin and Hilbe 2007, 60; McKillup 2012, 251–258).

## 5 Results

### 5.1 Geometric Morphometric Analyzes

Significant fluctuating asymmetry was identified in both maxillary and mandibular M1s (Tables 4 and 5). FA accounted for a relatively modest proportion of morphological variation (*circa* 14%–15%) with individual differences generating most of the observed variation. However, the Procrustes ANOVA process revealed that variation attributable to FA was approximately 26 times greater than that associated with error in the M^1^ (*F* = 26.411) and 35 times greater in the M_1_ (*F* = 35.943). Differences between replicates (i.e., intra‐observer error) accounted for 2.1% of variation in the M^1^ and 1.7% in the M_1_.

**TABLE 4:** M^1^ Procrustes ANOVA. FA is quantified through the interaction term of the procedure.

Effects	*df*	SS	MS	*R* ^2^	*F*	*p*
*ind*	153	3.530	0.023	0.833	5.854	0.781
*side*	1	0.011	0.011	0.002	2.862	0.003
*ind* × *side* (FA)	153	0.603	0.004	0.142	26.411	0.001
*error*	616	0.091	< 0.001	0.021		
Total	923	4.237				

**TABLE 5:** M_1_ Procrustes ANOVA. FA is quantified through the interaction term of the procedure.

Effects	*df*	SS	MS	*R* ^2^	*F*	*p*
*ind*	146	4.470	0.031	0.826	5.373	0.975
*side*	1	0.013	0.013	0.002	2.324	0.006
*ind* × *side* (FA)	146	0.832	0.006	0.154	35.943	0.001
*error*	588	0.093	< 0.001	0.017		
Total	881	5.409				

**FIGURE 1:**
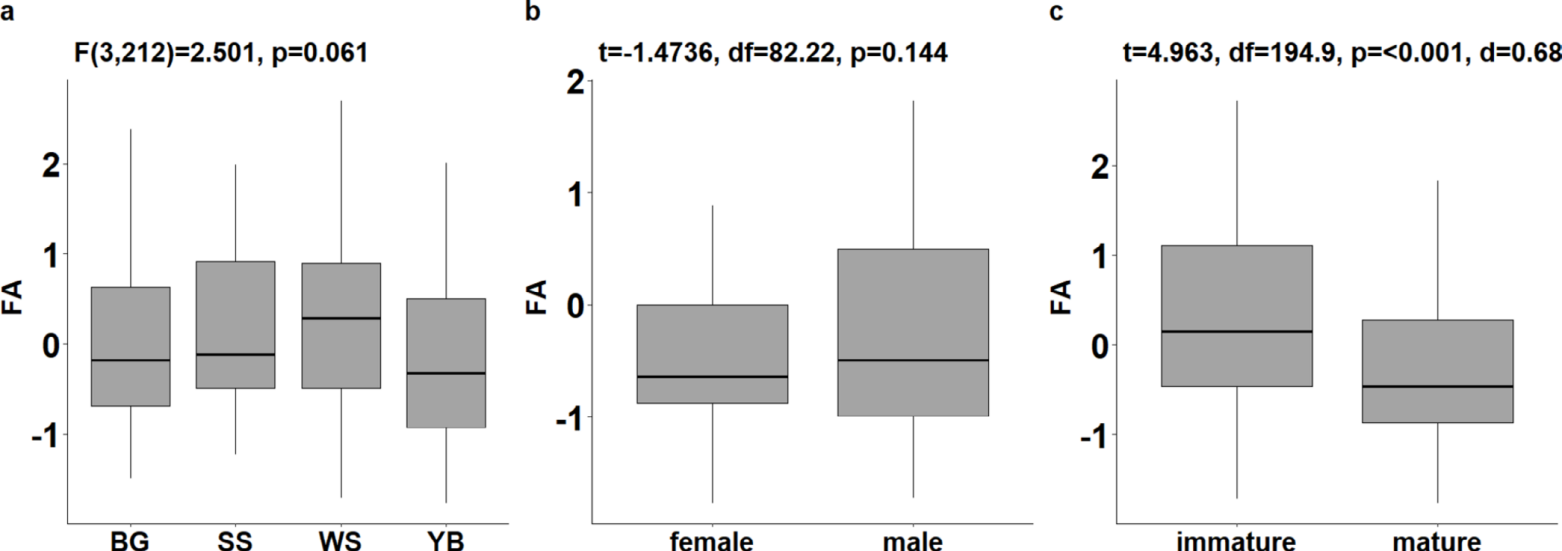
A comparison of FA scores between the sites of Black Gate (BG), South Shields (SS), Warwick (WS) and York Barbican (YB) (a), sexes (b), and skeletally immature and mature groups (c).

After individual composite FA scores were calculated, between group differences were evaluated. Following verification of underlying assumptions, an ANOVA procedure revealed that between‐site differences in FA scores were not significant (*F*(3,212) = 2.501, *p* = 0.061) (Figure 1a). Similarly, although male scores were more variable, a Welch's *t*‐test found no significant differences between sexes (*t* = −1.4736, *df* = 82.22, *p* = 0.144) (Figure 1b). However, there was a pronounced significant difference between skeletally mature and immature groups, with the latter associated with higher FA (*t* = 4.963, *df* = 194.9, *p* ≤ 0.001, Cohen's *d* = 0.68) (Figure 1c).

### 5.2 Demography and Paleopathology

**FIGURE 2:**
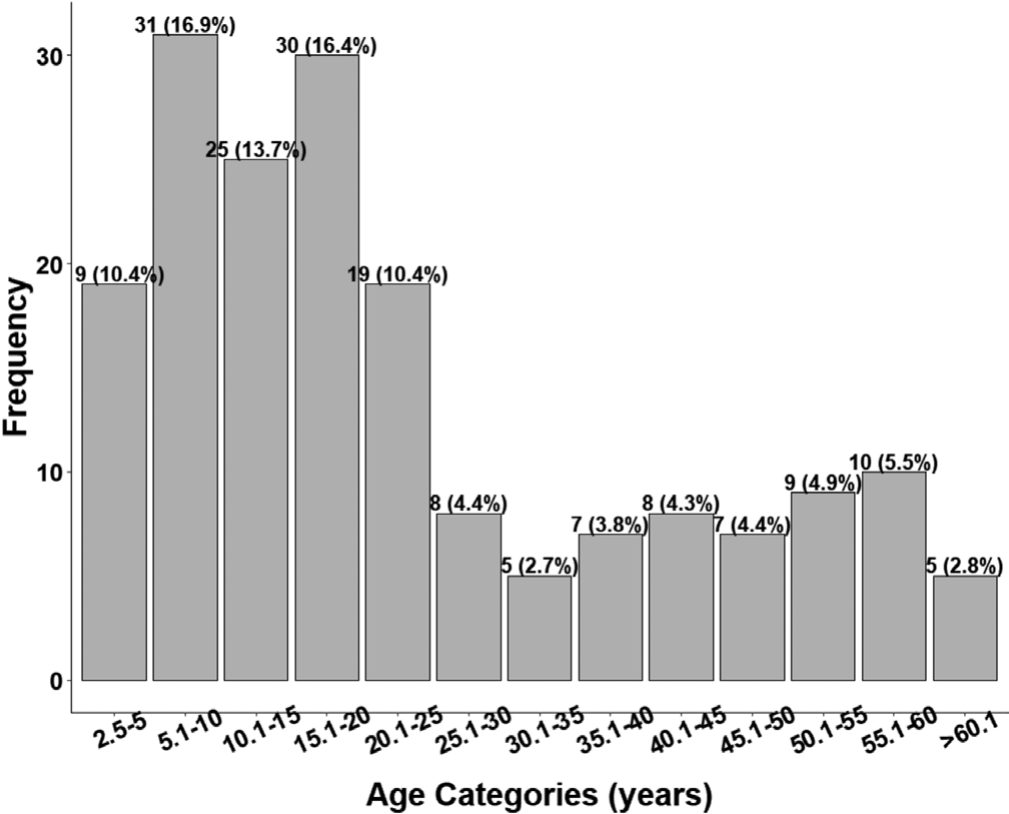
The age distribution of the sample.

There was a relatively high frequency of younger individuals in the sample (Figure 2 and Table 6). All individuals included survived the early‐life period, however, and the lowest age estimate was 2.5 years. For older individuals, mortality appeared to increase after *circa* 40 years of age. Although there was a slightly higher frequency of male skeletons (*n* = 49) than female (*n* = 36), this was not statistically significant and there was no sex bias between age cohorts or sites.

**TABLE 6:** A tabular summary of mortality in the overall sample as well as for each site.

Age categories	BG	SS	WS	YB	Combined
2.5–5	7 (9.9%)	4 (16.7%)	1 (4.0%)	7 (11.1%)	19 (10.4%)
5.1–10	10 (14.1%)	2 (8.3%)	13 (52.0%)	6 (9.5%)	31 (16.9%)
10.1–15	11 (15.5%)	3 (12.5%)	5 (20.0%)	6 (9.5%)	25 (13.7%)
15.1–20	7 (9.9%)	5 (20.8%)	2 (8.0%)	16 (25.4%)	30 (16.4%)
20.1–25	10 (14.1%)	0 (0.0%)	1 (4.0%)	8 (12.7%)	19 (10.4%)
25.1–30	6 (8.5%)	1 (4.2%)	0 (0.0%)	1 (1.6%)	8 (4.4%)
30.1–35	4 (5.6%)	0 (0.0%)	0 (0.0%)	1 (1.6%)	5 (2.7%)
35.1–40	4 (5.6%)	1 (4.2%)	0 (0.0%)	2 (3.2%)	7 (3.8%)
40.1–45	3 (4.2%)	2 (8.3%)	0 (0.0%)	3 (4.8%)	8 (4.3%)
45.1–50	3 (4.2%)	1 (4.2%)	1 (4.0%)	2 (3.2%)	7 (4.4%)
50.1–55	4 (5.6%)	1 (4.2%)	0 (0.0%)	4 (6.4%)	9 (4.9%)
55.1–60	1 (1.4%)	3 (12.5%)	1 (4.0%)	5 (7.9%)	10 (5.5%)
60.1–65	0 (0.0%)	1 (4.2%)	1 (4.0%)	0 (0.0%)	2 (1.1%)
65.1–70	0 (0.0%)	0 (0.0%)	0 (0.0%)	0 (0.0%)	0 (0.0%)
70.1–75	1 (1.4%)	0 (0.0%)	0 (0.0%)	1 (1.6%)	2 (1.1%)
75.1–80	0 (0.0%)	0 (0.0%)	0 (0.0%)	0 (0.0%)	0 (0.0%)
80.1–85	0 (0.0%)	0 (0.0%)	0 (0.0%)	0 (0.0%)	0 (0.0%)
85.1–90	0 (0.0%)	0 (0.0%)	0 (0.0%)	0 (0.0%)	0 (0.0%)
> 90	0 (0.0%)	0 (0.0%)	0 (0.0%)	1 (1.6%)	1 (0.5%)
Total	71	24	25	63	183

It was possible to assess the presence and absence of PNBF in 199 individuals and 1489 skeletal elements. When broken down and compared between cohorts (Tables 7 and 8), some clear trends emerged. The true prevalence of PNBF was higher in the later‐medieval collections of Warwick (TPR = 16.1%) and York Barbican (TPR = 12.5%) when compared to early‐medieval Black Gate (TPR = 7.2%) and post‐medieval South Shields (TPR = 10.2%). There was a similar rate of PNBF between mature (CPR = 30.4%, TPR = 10.5%) and immature individuals (CPR = 25.8%, TPR = 10.6%). However, when PNBF was present, there was a higher rate of individuals with active lesions in the immature cohort (CPR = 88.0%) compared to the mature (CPR = 51.6%). Similarly, there was a higher rate of bilateral cases among the immature individuals with PNBF (CPR = 82.4%) compared to mature skeletons with lesions (CPR = 69.0%).

**TABLE 7:** PNBF prevalence at each site. In brackets, the number of individuals/bones with PNBF is contrasted to the total number of individuals/bones.

Site	CPR of PNBF presence	TPR of PNBF presence
BG	19.2% (15/78)	7.2% (49/641)
SS	25.0% (7/28)	10.2% (18/177)
WS	24.0% (6/25)	16.1% (27/168)
YB	41.2% (28/68)	12.5% (63/503)

**TABLE 8:** The crude prevalence of active and bilateral PNBF among skeletally mature and immature individuals with lesions.

Maturity	CPR of active PNBF	CPR of bilateral PNBF
Immature	88.0% (22/25)	82.4% (14/17)
Mature	51.6% (16/31)	69.0% (20/29)

Regarding PD, all individuals in the sample (216) could be assessed and in total the alveolar regions associated with 1018 molars were observed. Again, higher frequencies of alveolar degeneration were seen among certain groups (Tables 9, 10, and 11). PD was most prevalent at South Shields (TPR = 51.5%), which was also the only site where the majority of skeletons (CPR = 58.8) had moderate‐to‐severe, rather than mild, signs of the condition. PD was relatively uncommon among immature skeletons (CPR = 14.4%, TPR = 10.9%). In contrast, PD was common in the mature cohort with most individuals (CPR = 73.2%) and molar tooth sockets (TPR = 50.2%) expressing some form of alveolar degeneration.

**TABLE 9:** PD prevalence at each site. In brackets, the number of individuals/molar sockets with PD is contrasted to the total number of individuals/molar sockets observed.

Site	CPR of PD presence	TPR of PD presence
BG	45.2% (38/84)	34.1% (117/343)
SS	51.5% (17/33)	51.5% (87/169)
WS	37.9% (11/29)	40.0% (48/120)
YB	44.3% (31/70)	32.4% (125/386)

**TABLE 10:** Severity of lesions in skeletons with PD.

Site	PD mild (CPR)	PD moderate (CPR)	PD severe (CPR)	Total
BG	33 (86.8%)	5 (13.2%)	0 (0.0%)	38
SS	7 (41.2%)	9 (52.9%)	1 (5.9%)	17
WS	9 (81.8%)	2 (18.2%)	0 (0.0%)	11
YB	27 (87.1%)	3 (9.7%)	1 (3.2%)	31

**TABLE 11:** PD prevalence in skeletally mature and immature cohorts. In brackets, the number of individuals/molar sockets with PD is contrasted to the total number of individuals/molar sockets observed.

Maturity	CPR of PD	TPR of PD
Immature	14.4% (15/104)	10.9% (37/341)
Mature	73.2% (82/112)	50.2% (340/677)

The above summarizes key findings. For further details of exploratory data analysis, refer to the Supporting Information.

### 5.3 Predicting Life‐Course Outcomes: Mortality Risk

Investigation of within‐group relationships between FA scores and age estimates detected a significant positive correlation between age‐at‐death and FA in the immature cohort. Interpretation of regression coefficients suggests that a one‐unit (i.e., one standard deviation; see Section 4: *Methods*) increase in FA scores predicts a 19.2% (95% CI [7.5%, 32.3%]) longer life within this cohort (Gelman and Hill 2007). The model, which explains a modest proportion of variation in estimated age‐at‐death (*F*(2,97) = 12.48, *p* ≤ 0.001, adj. *R* ^2^ = 0.19), also includes PD (Table 12). The positive coefficient associated with this lesion, implies PD presence predicts a 58.4% (95% CI [16.6%, 115.1%]) increase in length of life.

**TABLE 12:** Linear regression (*n* = 100) of log transformed estimated age‐at‐death in skeletally immature individuals on significant predictors (*F*(2,97) = 12.48, *p* ≤ 0.001, adj. *R* ^2^ = 0.19).

	Coefficient	95% CI	Std. error	*t*	*p*
Constant	2.095	1.978, 2.212	0.059	35.66	< 0.001
M1 FA score	0.176	0.072, 0.279	0.052	3.379	0.001
PD (present)	0.460	0.156, 0.764	0.153	3.002	0.003

In the regression of age‐at‐death in mature individuals (Table 13), PNBF, PD, FA scores and sex were all significant predictors and collectively accounted for a moderate proportion of variation (*F*(5,55) = 11.2, *p* ≤ 0.001, adj. *R* ^2^ = 0.46). Here, FA scores had a negative relationship with age‐at‐death, with a one‐unit increase predicting a 13.2% (95% CI [11.6%, 32.7%]) reduction in length of life. The procedure predicted that males had lives that were 19% (95% CI [6.8%, 30.4%]) shorter than females. While the presence of PD and PNBF had positive correlations with age‐at‐death, their significant interaction revealed that their co‐occurrence predicts a 32.5% (95% CI [1.3%, 53.8%]) shorter life.

**TABLE 13:** Linear regression (*n* = 61) of log transformed estimated age‐at‐death in skeletally mature individuals on significant predictors (*F*(5,55) = 11.2, *p* ≤ 0.001, adj. *R* ^2^ = 0.46).

	Coefficient	95% CI	Std. error	*t*	*p*
Constant	3.145	2.929, 3.361	0.108	29.154	< 0.001
PNBF (present)	0.640	0.304, 0.977	0.168	3.811	< 0.001
PD (present)	0.588	0.356, 0.819	0.116	5.089	< 0.001
M1 FA score	−0.154	−0.253, −0.056	0.049	−3.140	0.003
Sex (male)	−0.216	−0.363, −0.069	0.073	−2.944	0.005
PNBF × PD	−0.393	−0.774, −0.011	0.190	−2.063	0.044

### 5.4 Predicting Life‐Course Outcomes: Inflammatory Responses

**FIGURE 3:**
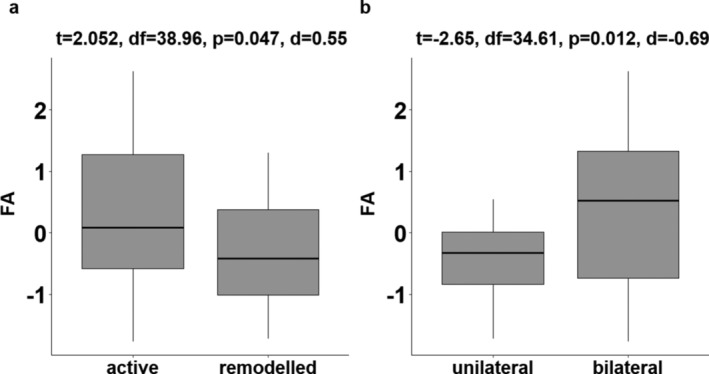
A comparison of FA scores between individuals with active versus remodeled lesions (a) and unilaterally and bilaterally distributed PNBF (b).

A logistic regression procedure revealed significant associations between periosteal lesions and age, sex and site (*χ* ^2^ = 164.36, *p* ≤ 0.001, McFadden's *R* ^2^ = 0.71) (Table 14); there was no significant interaction between age and site. The model indicated that males, older individuals and those from later‐medieval sites were significantly more likely to develop PNBF. In addition to this, although there was no significant difference in FA scores between individuals with and without PNBF (*t* = −0.674, *df* = 89.41, *p* = 0.502), among the individuals with lesions, a moderate difference between groups was detected with significantly higher FA observed in skeletons with active compared to remodeled lesions (*t* = 2.052, *df* = 38.96, *p* = 0.047, Cohen's *d* = 0.55) (Figure 3a). Despite this, logistic regression implies that skeletal maturity explains PNBF activity (*χ* ^2^ = 9.040, *p* = 0.003, McFadden's *R* ^2^ = 0.13) better than FA scores (Table 15). Similarly, FA was significantly higher in individuals who developed bilaterally distributed PNBF (*t* = −2.653, *df* = 34.61, *p* = 0.012, Cohen's *d* = −0.69) (Figure 3b) and, in this case, FA scores proved to be the best predictors in the regression model (*χ* ^2^ = 6.268, *p* = 0.012, McFadden's *R* ^2^ = 0.14) (Table 16). While the model only explains a modest proportion of variation, it implies that a one‐unit increase in FA predicts a 2.63 (95% CI [1.07, 6.48]) times higher risk of developing bilateral periosteal lesions.

**TABLE 14:** Logistic regression (*n* = 66) of PNBF absence (0) and presence (1) on age and site (*χ* ^2^ = 164.36, *p* ≤ 0.001, McFadden's *R* ^2^ = 0.71).

	Coefficient	95% CI	Std. error	*z*	*p*
Constant	−5.577	−9.089, −2.872	1.565	−3.564	< 0.001
Age	0.067	0.023, 0.121	0.025	2.726	0.006
Sex (male)	2.079	0.689, 3.758	0.772	2.693	0.007
Site (YB & WS)	1.627	0.383, 3.047	0.669	2.432	0.015

**TABLE 15:** Logistic regression (*n* = 56) of PNBF activity—that is, whether lesions were active (0) or healed (1)—on skeletal maturity (*χ* ^2^ = 9.040, *p* = 0.003, McFadden's *R* ^2^ = 0.13).

	Coefficient	95% CI	Std. error	*z*	*p*
Constant	−1.992	−3.433, −0.933	0.616	−3.237	0.001
Maturity (mature)	1.928	0.636, 3.508	0.713	2.705	0.007

**TABLE 16:** Logistic regression (*n* = 43) of PNBF distribution—that is, whether lesions were unilaterally (0) or bilaterally (1) expressed—on FA scores (*χ* ^2^ = 6.268, *p* = 0.012, McFadden's *R* ^2^ = 0.14).

	Coefficient	95% CI	Std. error	*z*	*p*
Constant	1.534	0.724, 2.610	0.463	3.313	< 0.001
M1 FA score	0.967	0.190, 2.010	0.451	2.144	0.032

PD in contrast did not have a discernible relationship with FA scores and there was little to no significant difference in scores between individuals with and without PD (*t* = −0.118, *df* = 26.52, *p* = 0.907). Regression procedures found that age, PNBF and site significantly predicted PD development (*χ* ^2^ = 81.541, *p* ≤ 0.001, McFadden's *R* ^2^ = 0.66) (Table 17). Specifically, it emerged that the risk of developing moderate‐to‐severe PD increased with age and was also higher at post‐medieval South Shields. Again, the interaction of age and site was not significant. Additionally, individuals with periosteal new bone formation are at a 5.11 (95% CI [1.36, 62.6]) times greater risk of developing moderate‐to‐severe PD.

**TABLE 17:** Logistic regression (*n* = 171) of PD presence on significant predictors when absent and mild cases were grouped together (0) and moderate and severe cases combined (1) (*χ* ^2^ = 81.541, *p* ≤ 0.001, McFadden's *R* ^2^ = 0.66).

	Coefficient	95% CI	Std. error	*z*	*p*
Constant	−8.421	−12.722, −5.662	1.743	−4.831	< 0.001
Age	0.099	0.056, 0.159	0.026	3.808	< 0.001
PNBF (present)	2.221	0.506, 4.389	0.958	2.319	0.020
Site (SS)	4.364	2.362, 7.151	1.180	3.698	< 0.001

## 6 Discussion

### 6.1 Contextual Influences and Intrinsic Frailty Differentials

PNBF and PD prevalence rates imply that environments play a key role in defining later‐life exposure to stressors. The significant association between periosteal lesions and the later‐medieval assemblages of Warwick and York Barbican could have several explanations. It may, for example, relate to a heightened prevalence of bacterial pathogens during the era and an impaired ability to resist them due to a series of famine events or, alternatively, the impact of hazardous labor practices and the concomitant increase in fracture risk (Harley 1958, 18; John 1997; Slavin 2013). In contrast, it is believed that the elevated prevalence of moderate‐to‐severe PD in the South Shields sample (CPR = 58.8) relates to changes in diet (e.g., increasing consumption of sugar) and the advent of tobacco‐smoking during the post‐medieval period, both of which promoted poor oral health and alveolar degeneration (Davies‐Barrett and Inskip 2024; Gaskell 1836, 118–119; Lockhart et al. 2012; 2536; Newman 2016, 60; Palubeckaite et al. 2006, 362).

Intrinsic differentials in frailty also shaped morbidity and mortality risk. The positive correlations identified through regressions tests between age and both PNBF and PD presence demonstrates that the longer an individual is exposed to a deleterious environment, the greater the chance of being affected by stressors that provoke degenerative skeletal changes. Regression procedures also revealed that males were significantly more likely to develop periosteal lesions and were predicted to live 19% (95% CI [6.8%, 30.4%]) shorter lives than females. These findings imply that as individuals become older resilience diminishes and reiterate well‐established sex differentials in morbidity and mortality (DeWitte 2010; Stinson 1985). Together they suggest that intrinsic frailty (i.e., due to age or sex) influences the impact of contextually defined stressors.

### 6.2 FA, Inflammatory Lesions and Mortality

The GM assessment of M1 occlusal FA is a reliable and an interrogatively useful approach (Wigley, Stillman, and Craig‐Atkins 2024). It was found that intra‐observer error only accounted for a marginal proportion of overall variation (2.1% in the M^1^ and 1.7% in the M_1_) and variation due to FA was between 26 and 35 times greater than error. This is crucial as fluctuating asymmetry is a subtle marker of stress and is easily obscured by recorder inconsistency. Moreover, the level of error is comparable with recent research, such as McPherson et al. (2024) who reported an intra‐observer error rate of 1.8% in deciduous dental FA. Regarding the utility of the approach, the most illuminating findings came from within‐group analyzes of estimated age‐at‐death which included individual FA scores in conjunction with inflammatory lesions.

Within the immature cohort, age‐at‐death had a significant positive correlation with FA scores and PD presence. To illustrate, a one unit increase in FA predicted a 19.2% (95% CI [7.5%, 32.3%]) longer life. As FA and dental defects are generally associated with elevated morbidity and mortality risk (e.g., Dewitte and Wood 2008; Temple 2014; Yaussy 2024) and PD is a pathological condition (Caruso and Nikita 2024, 13; DeWitte and Bekvalac 2010; Larsen 1997, 77; Ogden 2008), this was unexpected. It does, however, suggest that stressors can be linked to positive outcomes (i.e., a longer life). As M1 FA is a proxy for early‐life stress, it is proposed that for those individuals who survived early life, perturbations experienced during this period of elevated plasticity were critical in shaping phenotypic shifts which enhanced survivorship during later development (i.e., in childhood and adolescence). The positive contribution made by PD presence to the regression model is more challenging to interpret. It is not inconsistent with clinical research which has suggested that inflammatory responses, while eventually deleterious, can initially be helpful in containing the spread of perturbations to health such as bacterial and viral pathogens (Gustine and Jones 2021; Oishi and Manabe 2018; Tan, Komarasamy, and Balasubramaniam 2021; Zietek and Rath 2016). However, it could also reflect the fact that periodontal lesions are more likely to occur as individuals become older, as shown by the regression of lesion presence on age estimates and other factors.

Quite different results were detected in the regression of age‐at‐death in the mature group. It was found that a one unit increase in FA scores predicts a substantial reduction in length of life (i.e., by 13.2% (95% CI [11.6%, 32.7%])), implicating early‐life stress in increased mortality risk during later‐life for individuals that survived into maturity. That is, stressors experienced during early life appear to be associated with heterogeneity in frailty. While this appears to be at odds with the pattern identified in the immature cohort, it does align with past research. For example, Yaussy (2024) found that higher craniofacial FA (another marker of developmental stress) was associated with earlier age‐at‐death in adults (i.e., skeletally mature individuals for whom sex could be estimated). It was also noted here that the combined presence of PNBF and PD in mature individuals predicts a shorter life (i.e., by 32.5% (95% CI [1.3%, 53.8%])). Again, while this seemingly contradicts the results of analyzes conducted on the immature group, it is congruent with the supposition that elevated systemic inflammation, being energetically costly and disruptive to normal somatic functioning, is ultimately harmful (Gustine and Jones 2021; Tan, Komarasamy, and Balasubramaniam 2021; Wells 2016, 141–142).

Thus far, it has been proposed that for individuals who survive past early life, stress experienced in this period is implicated in phenotypic programming that initially promotes resilience during subsequent development. The significantly higher FA scores among the immature cohort (*t* = 4.963, *df* = 194.9, *p* ≤ 0.001, Cohen's *d* = 0.68) as well as the negative coefficient associated with FA scores in the regression of age in the mature cohort do, however, strongly suggest that, despite possible immediate/short‐term benefits (discussed below), elevated early‐life stress is associated with increased mortality risk and variation in frailty for mature individuals. Similarly, the higher prevalence of active (CPR = 88.0%) and bilateral PNBF (CPR = 82.4%) among the immature individuals with PNBF implies that unresolved and diffuse inflammation are linked to reduced survivorship. These findings generate a complex picture of the interactions between early‐life stress, physiological phenotype, and mortality risk.

### 6.3 Shifts in Physiological Phenotype?

Further connections can be drawn between FA and inflammatory lesions. Specifically, FA scores were significant predictors of whether periosteal lesions had a unilateral or bilateral distribution (*χ* ^2^ = 6.268, *p* = 0.012, McFadden's *R* ^2^ = 0.14) with the latter lesions associated with significantly higher FA (*t* = −2.653, *df* = 34.61, *p* = 0.012). Significantly higher FA scores were also observed among individuals with active PNBF (*t* = 2.052, *df* = 38.96, *p* = 0.047), although this was complicated by the greater frequency of immature skeletons with active lesions due to their generally elevated M1 FA. Based on these findings, it is suggested that heightened early‐life stress increases the chances of developing systemic inflammation which either took longer to resolve or was less likely to do so in response to later‐life perturbations. Given periosteal lesions, especially when diffuse and symmetrically distributed, are frequently associated with an inflammatory response to infection (Roberts and Buikstra 2019; Roberts 2019; Weston 2008, 49), the results of this project may implicate early‐life stress in reduced immune competence.

As a counterpoint to this interpretation, it might be argued that connections between FA and PNBF evidence a predisposed frailty across the life‐course and the impact that has on the likelihood of manifesting stressors skeletally, rather than the influence of stressors endured earlier in life on later experience (DeWitte 2010, 8; DeWitte and Stojanowski 2015, 399; Vaupel 1988, 277; Wood et al. 1992, 357). Although that is perhaps the most parsimonious explanation, experimental and clinical research implies it is not the most probable. It has been demonstrated that exposure to a physiological stressor can lead to systemic changes. This may include a shift in physiological phenotype which increases the chances of later developing a suite of inflammatory lesions, as well as more aggressive and diffuse inflammatory responses (e.g., Crespo et al. 2016; Tan, Komarasamy, and Balasubramaniam 2021). Associations between early‐life stress and inflammation might also be questioned due to the absence of a discernible comorbidity between FA and PD. Possibly due to the localized nature of periodontal disease, its development is more dependent upon dietary factors and oral health than systemic changes in physiology (Caruso and Nikita 2024, 13; Davies‐Barrett and Inskip 2024; Larsen 1997, 77). Alternatively, the method employed to assess PD here may not have been optimal and poorly captured in vivo disease manifestation, potentially obscuring links between FA and PD (Bertl et al. 2020; Ogden 2008).

Although the evidence must be treated cautiously, plausible links between skeletal proxies for early‐life stress (i.e., M1 FA) and aggressive and/or systemic inflammatory responses (i.e., active and bilateral PNBF) in later‐life have been identified. It appears that while contextual factors are important in determining exposure to risk factors (e.g., infectious agents) that lead to skeletal inflammation, early‐life stress likely impacts the characteristics of inflammatory responses to later‐life perturbations. Stressors experienced during the critical early‐life period are therefore implicated in durable physiological shifts, specifically the development of a “hyperinflammatory” physiology in which aggressive inflammation is more common (e.g., Tan, Komarasamy, and Balasubramaniam 2021, 3). It is doubted that such a shift is beneficial in the long term, as is evident from the prediction of a reduced length of life for mature individuals with the combined presence of PNBF and PD. However, research demonstrates inflammation elicits a mix of outcomes (Gustine and Jones 2021; Oishi and Manabe 2018; Zietek and Rath 2016). Tan, Komarasamy, and Balasubramaniam (2021), for example, reported that while in the long‐term stimulation of multiple inflammatory pathways increases the risk of tissue damage and multi‐organ failure, it can initially contain pathogen spread and enhance survivorship.

### 6.4 Adaptive Significance of Stress‐Induced Variation

The results presented here have clear implications regarding the adaptive importance of stress‐induced phenotypic shifts. Developmental stress appears to provoke phenotypic alterations (possibly shifts in inflammatory response) which enhance the likelihood of survival during immaturity at the expense of later‐life outcomes in morbidity and mortality.

It could be argued that the pattern of life‐course experiences/outcomes identified here represent a Predictive Adaptive Response with a mismatch between developmental and later‐life environment. Environmental mismatches result from faulty “forecasts” of conditions. Somatic plasticity decreases following early life, leaving individuals insufficiently flexible to re‐adapt. Mismatches in early and later‐life conditions can therefore render maladaptive a formerly beneficial PAR (Bateson et al. 2004; Bateson, Gluckman, and Hanson 2014). Thus, the paradoxical positive correlation between M1 FA scores and immature age‐at‐death, but the higher scores observed among this cohort in comparison to the mature group, may be accounted for by unexpected environmental changes diminishing the value of unmodifiable shifts in phenotype. It can, however, be questioned to what extent PARs, which rely on an accurate long‐range forecast of conditions (Bateson, Gluckman, and Hanson 2014, 2358; Wells 2010, 4), are beneficial for human populations. Humans habitually manipulate and alter environments in processes of construction and reconstruction, and the value of long‐term predictive adaptations is doubted (Fuentes, Wyczalkowski, and MacKinnon 2010; Lu et al. 2019; Prince‐Buitenhuys and Bartelink 2021; Wells 2012, 232).

Instead, we argue the findings and the interpretations presented here more naturally align with the Thrifty Phenotype hypothesis. Hypothesis 1 (see Section 2: *Background*) predicted M1 FA would be linked to patterns in stress‐marker prevalence and/or age‐at‐death that implied shifts in phenotype which were initially adaptive would be later associated with increased morbidity and/or earlier age‐at‐death. This articulated the expectations of the Thrifty Phenotype hypothesis which proposes that the allocation of somatic resources during development promotes immediate/short‐term survival. Where resources are scarce this comes at the expense of constrained investment in other physiological systems and dysregulation in later‐life, increasing morbidity and mortality risk (e.g., Hales and Barker 2013; Pomeroy et al. 2012). The Thrifty Phenotype hypothesis is highly consistent with the supposition advocated here: that early‐life stress (inferred through M1 FA scores) was associated with both enhanced survivorship during development (i.e., whilst skeletally immature), but also with later pathological inflammation and shorter length of life. Such an outcome is likely adaptive in harsh environments, such as those experienced by the historical populations sampled here, where access to nutritional resources is unreliable (e.g., due to famine or seasonal availability) and disease burden is high. In such conditions, relatively few individuals reach old age, regardless of how well physiological systems are regulated and maintained for long‐term functioning. The chances of achieving success from an evolutionary perspective (i.e., producing offspring) are therefore maximized by investing resources in robust responses to immediate physiological perturbations (e.g., through hyperinflammatory responses) to increase the likelihood attaining somatic maturity, even if that is at the expense of increased later‐life morbidity and mortality risk (Ellis and Del Guidice 2019, 116; Metcalfe and Monaghan 2001, 257; Wells 2016, 131–144).

Returning to the question posed at the beginning of the paper, it appears that adverse experiences which are not immediately fatal may in fact encourage adaptations that are beneficial. These phenotypic alterations are, however, seemingly only adaptive in the short‐term and likely have a deleterious impact on future morbidity and length of life. So, it may be more accurate to rephrase the popular saying to something along the lines of “What doesn't kill you makes you stronger for a while, but you pay for it later.”

### 6.5 Sampling Considerations and Further Research

The skeletons analyzed here were drawn from different collections. This enhanced sample size and statistical power. However, it could have introduced variation into the analysis that distorted results, especially when exploring mortality patterns and lesions that are age‐progressive. Despite this, the results of age estimation suggested the overall sample and the subsets drawn from each collection broadly approximated a typical pre‐modern living population with an attritional mortality profile (Chamberlain 2000; Gowland and Chamberlain 2005, 146). This is congruent with previous analyzes involving complete assemblages (e.g., Mahoney Swales 2019; McIntyre and Bruce 2010; Raynor, McCarthy, and Clough 2011). Moreover, the interaction of site and age was not significant in the logistic regression procedures employed to predict PNBF and PD presence, suggesting that analyzes had not been confounded by differences in the proportion of younger/older individuals at each site and the age‐progressive nature of these pathologies. The trends of mortality identified in this paper are unlikely to be influenced by catastrophic events and inferences drawn from analyzes are likely applicable to other samples and living populations.

Due to the methodology employed certain age‐related biases were identified. As M1s do not erupt until approximately the sixth year of life and sampling did not involve invasive and destructive techniques to remove unerupted teeth from the alveolar processes, relatively few infants and younger children were assessed (i.e., only those with M1s freed from alveolar crypts by post‐mortem breakage). It is not believed that FA had yet to manifest in any of these individuals as the youngest age estimate (2.5 years) is beyond the point at which the spatial patterning of M1 occlusal features is fixed through the mineralization of developmental signaling centers (i.e., around the end of the first postnatal year). Moreover, the maximal diameter (i.e., the occlusal outline) of a tooth is approximately one‐third to halfway between the occlusal surface and the cemento‐enamel junction (Benazzi et al. 2011, 349). As this region mineralizes by the end of the second postnatal year (Reid and Dean 2006, 334), the M1s of even the youngest individual were sufficiently well‐developed for the outline analysis employed here (Wigley, Stillman, and Craig‐Atkins 2024). Conversely, as skeletons were selected based on the availability of two observable and relatively unworn antimeric M1s, the oldest individuals are likely underrepresented due to age‐progressive factors such as dental wear and ante‐mortem tooth loss. In fact, only five individuals were associated with age estimates > 60 years. Potentially this may have obscured patterns in mortality.

Finally, despite the substantial disparities in social, cultural, and economic conditions associated with each assemblage, between‐site differences in FA scores were not statistically significant (*F*(3,212) = 2.501, *p* = 0.061). Moreover, well‐established differentials in frailty were not observed when FA was contrasted between sexes (*t* = −1.4736, *df* = 82.22, *p* = 0.144). Given that offspring are maternally‐dependent during early‐life, it is proposed that external stressors and intrinsic differentials were mitigated during this critical period. Variation in M1 FA scores may therefore reflect differences in maternal condition and offer an opportunity for further research to explore inter‐generational health in past populations (Wigley, Stillman, and Craig‐Atkins 2024).

## 7 Conclusion

This paper has tested the theoretical expectations associated with the Thrifty Phenotype and Predictive Adaptive Response hypotheses (e.g., Bateson, Gluckman, and Hanson 2014; Hales and Barker 2013). Four main findings can be drawn from the data. First, environmental factors play an important role in stress exposure and responses to stressors can be mitigated or magnified by age and sex differentials in frailty. Second, a contradictory relationship between early‐life stress, inflammation, and mortality risk was identified. Potentially, for those individuals who survived early life, elevated early‐life stress and a predisposition towards aggressive and systemic inflammatory responses initially promote survival, but in the long run are associated with increased frailty and mortality risk. Third, it is proposed that early‐life stress contributes to durable shifts in physiology and influences the development of a hyperinflammatory physiology in later‐life. Finally, these findings, while not entirely inconsistent with the expectations of the PAR model, align better with Hales and Barker's (1992, 2001, 2013) Thrifty Phenotype hypothesis. This posits that stress‐induced phenotypic alterations eventually lead to increased morbidity and mortality risk which, while not definitive, can have a substantial impact on life‐course outcomes.

## Author Contributions

**B. R. Wigley:** conceptualization (lead), data curation (lead), formal analysis (lead), funding acquisition (equal), investigation (lead), methodology (lead), project administration (equal), writing – original draft (lead), writing – review and editing (lead). **E. C. Stillman:** conceptualization (equal), data curation (supporting), formal analysis (supporting), funding acquisition (equal), investigation (supporting), methodology (supporting), project administration (equal), supervision (equal), writing – review and editing (equal). **E. Craig‐Atkins:** conceptualization (equal), data curation (supporting), formal analysis (supporting), funding acquisition (equal), investigation (supporting), methodology (supporting), project administration (equal), supervision (equal), writing – review and editing (equal).

## Conflicts of Interest

The authors declare no conflicts of interest.

## Supporting information


**Data S1** Supporting Information.

## Data Availability

The data analyzed in the paper is stored in ORDA, the University of Sheffield's online data repository, and can be found at: https://doi.org/10.15131/shef.data.26788552.v1.

## References

[ajpa70005-bib-0001] Adams, D. C. , M. L. Collyer , A. Kaliontzopoulou , and E. Baken . 2023. “Geomorph: Software for Geometric Morphometric Analyses.” *R Package Version 4.0.6*. https://cran.r‐project.org/package=geomorph.

[ajpa70005-bib-0002] Agarwal, S. C. 2016. “Bone Morphologies and Histories: Life Course Approaches in Bioarchaeology.” Yearbook of Physical Anthropology 159: 130–149. 10.1002/ajpa.22905 26808102

[ajpa70005-bib-0003] AlQahtani, S. , M. P. Hector , and H. M. Liversidge . 2010. “Brief Communication: The London Atlas of Human Tooth Development and Eruption.” American Journal of Physical Anthropology 142: 481–490. 20310064 10.1002/ajpa.21258

[ajpa70005-bib-0004] Antoine, D. , and S. Hillson . 2016. “Enamel Structure and Properties.” In A Companion to Dental Anthropology, edited by J. D. Irish and G. R. Scott , First ed., 223–243. London: Wiley.

[ajpa70005-bib-0005] Barker, D. J. P. 2012. “The Developmental Origins of Health and Disease.” Public Health 126: 186. 10.1016/j.puhe.2011.11.014 22325676

[ajpa70005-bib-0006] Barker, D. J. P. , and C. Osmond . 1986. “Infant Mortality, Childhood Nutrition and Ischaemic Heart Disease in England and Wales.” Lancet 1, no. 8489: 1077–1081. 2871345 10.1016/s0140-6736(86)91340-1

[ajpa70005-bib-0007] Barrett, J. H. , A. M. Locker , and C. M. Roberts . 2004. “Dark Age Economics Revisited.” Antiquity 78: 618–636.

[ajpa70005-bib-0008] Bateson, P. , D. Barker , T. Clutton‐Brock , et al. 2004. “Developmental Plasticity and Human Health.” Nature 430: 419–421. 15269759 10.1038/nature02725

[ajpa70005-bib-0009] Bateson, P. , P. D. Gluckman , and M. A. Hanson . 2014. “The Biology of Developmental Plasticity and the Predictive Adaptive Response Hypothesis.” Journal of Physiology 592, no. 11: 2357–2368. 24882817 10.1113/jphysiol.2014.271460 PMC4048093

[ajpa70005-bib-0010] Benazzi, S. , M. Coquerelle , L. Fiorenza , F. Bookstein , S. Katina , and O. Kullmer . 2011. “Comparison of Dental Measurement Systems for Taxonomic Assignment of First Molars.” American Journal of Physical Anthropology 144: 342–354. 21302262 10.1002/ajpa.21409

[ajpa70005-bib-0011] Bertl, K. , S. Tang , T. Rybaczek , et al. 2020. “Prevalence and Severity of Periodontal Disease in a Historical Austrian Population.” Journal of Periodontal Research 55, no. 6: 931–945. 32658361 10.1111/jre.12785 PMC7689777

[ajpa70005-bib-0012] Bleker, L. S. , S. R. de Rooij , R. C. Painter , A. C. J. Ravelli , and T. J. Roseboom . 2021. “Cohort Profile: The Dutch Famine Birth Cohort (DFBC) – A Prospective Birth Cohort Study in The Netherlands.” BMJ Open 11: e042078. 10.1136/bmjopen-2020-042078 PMC7934722 33664071

[ajpa70005-bib-0013] Boldsen, J. l. , G. R. Milner , L. W. Konigsberg , and J. W. Wood . 2002. “Transition Analysis. A New Method for Estimating Age From Skeletons.” In Paleodemography. Age Distributions From Skeletal Samples, edited by R. D. Hoppa and J. W. Vaupel , 73–106. Cambridge: Cambridge University Press.

[ajpa70005-bib-0014] Bookstein, F. L. 1991. “Morphometric Tools for Landmark Data.” In Geometry and Biology. Cambridge: Cambridge University Press.

[ajpa70005-bib-0015] Bruce, G. 2003. “The Barbican Centre, York. *OSA Report No: Osa03ev08*. Report on Archaeological Evaluation.” *On Site Archaeology* .

[ajpa70005-bib-0016] Buikstra, J. E. , and D. H. Ubelaker . 1994. Standards for Data Collection From Human Skeletal Remains. Arkansas: Twelfth Printing 2010.

[ajpa70005-bib-0017] Caruso, A. , and E. Nikita . 2024. “Dental Diseases and Dental Wear as a Proxy for Dietary Patterns in Hellenistic‐Early Roman Menainon, Sicily.” International Journal of Paleopathology 44: 10–19. 38039701 10.1016/j.ijpp.2023.11.002

[ajpa70005-bib-0018] Chamberlain, A. 2000. “Problems and Prospects in Palaeodemography.” In Human Osteology in Archaeology and Forensic Science, edited by M. Cox and S. Mays , 101–106. Cambridge: Cambridge University Press.

[ajpa70005-bib-0019] Chipirliu, O. , M. V. Craciun , and M. N. Matei . 2024. “Comparative Clinical Study on Periodontal Health Status and Early Diagnosis of Periodontal Diseases Quantified Through Clinical Periodontal Indices on a Group of Children and Adolescents With and Without Cardiovascular Diseases.” Pediatric Reports 16: 1–20. 10.3390/pediatric16010001 PMC10801528 38251310

[ajpa70005-bib-0020] Collyer, M. L. , and D. C. Adams . 2018. “RRPP: An R Package for Fitting Linear Models to High‐Dimensional Data Using Residual Randomization.” Methods in Ecology and Evolution 9: 1772–1779.

[ajpa70005-bib-0021] Collyer, M. L. , and D. C. Adams . 2024. “Interrogating Random and Systematic Measurement Error in Morphometric Data.” Evolutionary Biology 51: 179–207.

[ajpa70005-bib-0022] Crespo, F. A. 2021. “Reconstructing Immune Competence in Skeletal Samples. A Theoretical and Methodological Approach.” In Theoretical Approaches in Bioarchaeology, edited by C. M. Cheverko , J. R. Prince‐Buitenhuys , and M. Hubbe , 76–92. Oxford: Routeledge.

[ajpa70005-bib-0023] Crespo, F. A. , C. K. Klaes , A. E. Switala , and S. N. DeWitte . 2016. “Do Leprosy and Tuberculosis Generate a Systemic Inflammatory Shift? Setting the Ground for a New Dialogue Between Experimental Immunology and Bioarchaeology.” American Journal of Physical Anthropology 162: 143–156. 27704524 10.1002/ajpa.23104

[ajpa70005-bib-0024] Davies‐Barrett, A. , and S. Inskip . 2024. “Who Smokes Anymore? Documentary, Archaeological and Osteological Evidence for Tobacco Consumption and Its Relationship to Social Identity in Industrial England, 1700‐1850.” In The Material Body. Embodiment, History and Archaeology in Industrializing England, edited by E. Craig‐Atkins and K. Harvey , 133–169. Manchester: Manchester University Press.

[ajpa70005-bib-0025] DeWitte, S. N. 2010. “Sex Differentials in Frailty in Medieval England.” American Journal of Physical Anthropology 143, no. 2: 285–297. 20853482 10.1002/ajpa.21316 PMC3097521

[ajpa70005-bib-0027] DeWitte, S. N. , and J. Bekvalac . 2010. “Oral Health and Frailty in the Medieval English Cemetery of St Mary Graces.” American Journal of Physical Anthropology 142: 341–354. 19927365 10.1002/ajpa.21228 PMC3094918

[ajpa70005-bib-0028] DeWitte, S. N. , and J. Bekvalac . 2011. “The Association Between Periodontal Disease and Periosteal Lesions in the St. Mary Graces Cemetery, London, England A.D. 1350‐1538.” American Journal of Physical Anthropology 146: 609–618. 21997205 10.1002/ajpa.21622

[ajpa70005-bib-0029] DeWitte, S. N. , and C. Stojanowski . 2015. “The Osteological Paradox 20 Years Later: Past Perspectives, Future Directions.” Journal of Archaeological Research 23: 397–450.

[ajpa70005-bib-0030] DeWitte, S. N. , and J. W. Wood . 2008. “Selectivity of Black Death Mortality With Respect to Preexisting Health.” Proceedings of the National Academy of Sciences of the United States of America 105, no. 5: 1436–1441. 18227518 10.1073/pnas.0705460105 PMC2234162

[ajpa70005-bib-0031] Dryden, I. L. , and K. V. Mardia . 2016. Statistical Shape Analysis, With Applications in R. Second ed. Chichester: Wiley.

[ajpa70005-bib-0032] Ellis, B. J. , and M. Del Guidice . 2019. “Developmental Adaptation to Stress: An Evolutionary Perspective.” Annual Review of Psychology 70: 111–139. 10.1146/annurev-psych-122216-011732 30125133

[ajpa70005-bib-0033] Escós, J. M. , C. L. Alados , F. I. Pugnaire , J. Puigdefábregas , and J. M. Emlen . 2000. “Stress Resistance Strategy in Arid Land Shrub: Interaction Between Developmental Instability and Fractal Dimension.” Journal of Arid Environments 45: 325–336.

[ajpa70005-bib-0034] Ferembach, D. , I. Schwidetzky , and M. Stloukal . 1980. “Recommendations for Age and Sex Diagnoses of Skeletons.” Journal of Human Evolution 9: 517–549.

[ajpa70005-bib-0035] Fuentes, A. , M. A. Wyczalkowski , and K. C. MacKinnon . 2010. “Niche Construction Through Cooperation: A Nonlinear Dynamics Contribution to Modeling Facets of the Evolutionary History in the Genus Homo.” Current Anthropology 51, no. 3: 435–444.

[ajpa70005-bib-0036] Gaskell, P. 1836. Artisans and Machinery: The Moral and Physical Condition of the Manufacturing Population Considered With Mechanical Substitutes for Human Labour. London: John W. Parker.

[ajpa70005-bib-0037] Gelman, A. , and J. Hill . 2007. Data Analysis Using Regression and Multilevel/Hierarchical Models. Cambridge: Cambridge University Press.

[ajpa70005-bib-0038] Gethin, B. n.d. *Interim Report on Archaeological Investigations at the Site of St Lawrence's Church, 5 Stratford Road, Warwick, Warwickshire*. Unpublished Report.

[ajpa70005-bib-0039] Getz, S. M. 2020. “The Use of Transition Analysis in Skeletal Age Estimation.” Forensic Science 2: e1378.

[ajpa70005-bib-0040] Gluckman, P. D. , M. A. Hanson , and A. S. Beedle . 2007. “Early Life Events and Their Consequences for Later Disease: A Life History and Evolutionary Perspective.” American Journal of Human Biology 19: 1–19. 17160980 10.1002/ajhb.20590

[ajpa70005-bib-0041] Gluckman, P. D. , M. A. Hanson , and T. Buklijas . 2010. “A Conceptual Framework for the Developmental Origins of Health and Disease.” Journal of Developmental Origins of Health and Disease 1, no. 1: 6–18. 25142928 10.1017/S2040174409990171

[ajpa70005-bib-0042] Goldberg, J. 2019. “Making the House a Home in Later Medieval York.” Journal of Medieval History 45, no. 2: 162–180.

[ajpa70005-bib-0044] Gowland, R. L. , and J. L. Caldwell . 2023. “The Developmental Origins of Health and Disease: Implications for Paleopathology.” In The Routledge Handbook of Paleopathology, edited by A. L. Grauer , 520–540. London: Routledge.

[ajpa70005-bib-0045] Gowland, R. L. , and A. T. Chamberlain . 2005. “Detecting Plague: Palaeodemographic Characterisation of a Catastrophic Death Assemblage.” Antiquity 79, no. 303: 146–157.

[ajpa70005-bib-0046] Graham, J. H. , and B. Ozener . 2016. “Fluctuating Asymmetry of Human Populations: A Review.” Symmetry 8: 154.

[ajpa70005-bib-0047] Graham, J. H. , S. Raz , H. Hagit , and E. Nevo . 2010. “Fluctuating Asymmetry: Methods, Theory and Applications.” Symmetry 2: 466–495.

[ajpa70005-bib-0048] Grus, J. 2015. Data Science From Scratch First Principles With Python. London: O'Reilly.

[ajpa70005-bib-0049] Gustine, J. N. , and D. Jones . 2021. “Immunopathology of Hyperinflammation in COVID‐19.” American Journal of Pathology 191, no. 1: 4–17. 32919977 10.1016/j.ajpath.2020.08.009 PMC7484812

[ajpa70005-bib-0050] Hales, C. N. , and D. J. P. Barker . 1992. “Type 2 (Non‐Insulin‐Dependent) Diabetes Mellitus: The Thrifty Phenotype Hypothesis.” Diabetologia 35: 595–601. 1644236 10.1007/BF00400248

[ajpa70005-bib-0051] Hales, C. N. , and D. J. P. Barker . 2001. “The Thrifty Phenotype Hypothesis.” British Medical Bulletin 60: 5–20. 11809615 10.1093/bmb/60.1.5

[ajpa70005-bib-0052] Hales, C. N. , and D. J. P. Barker . 2013. “Type 2 (Non‐Insulin‐Dependent) Diabetes Mellitus: The Thrifty Phenotype Hypothesis.” International Journal of Epidemiology 42: 1215–1222. 24159065 10.1093/ije/dyt133

[ajpa70005-bib-0053] Hardin, J. W. , and J. M. Hilbe . 2007. Generalized Linear Models and Extensions. College Drive: Stata Press.

[ajpa70005-bib-0054] Harley, J. B. 1958. “Population Trends and Agricultural Developments From the Warwickshire Hundred Rolls of 1279.” Economic History Review 11, no. 1: 8–18.

[ajpa70005-bib-0055] Jernvall, J. , and H.‐S. Jung . 2000. “Genotype, Phenotype, and Developmental Biology of Molar Tooth Characters.” Yearbook of Physical Anthropology 43: 171–190. 10.1002/1096-8644(2000)43:31+<171::aid-ajpa6>3.0.co;2-3 11123840

[ajpa70005-bib-0056] John, T. 1997. “Population Change in Medieval Warwickshire: Domesday Book to the Hundred Rolls of 1279‐1280.” Local Population Studies 59: 41–53.

[ajpa70005-bib-0057] Kenessey, D. E. , C. M. Stojanowski , and K. S. Paul . 2024. “Evaluating Predictions of the Patterning Cascade Model of Crown Morphogenesis in the Human Lower Mixed and Permanent Dentition.” PLoS One 19, no. 6: e0304455. 38935640 10.1371/journal.pone.0304455 PMC11210800

[ajpa70005-bib-0058] Klingenberg, C. P. 2015. “Analysing Fluctuating Asymmetry With Geometric Morphometrics: Concepts, Methods, and Applications.” Symmetry 7: 843–934.

[ajpa70005-bib-0059] Klingenberg, C. P. , and G. S. McIntyre . 1998. “Geometric Morphometrics of Developmental Instability: Analyzing Patterns of Fluctuating Asymmetry With Procrustes Methods.” Evolution; International Journal of Organic Evolution 52, no. 5: 1363–1375. 28565401 10.1111/j.1558-5646.1998.tb02018.x

[ajpa70005-bib-0060] Koutsochristou, V. , A. Zellos , K. Dimakou , et al. 2015. “Dental Caries and Periodontal Disease in Children and Adolescents With Inflammatory Bowel Disease: A Case‐Control Study.” Inflammatory Bowel Diseases 21, no. 8: 1839–1846. 25985243 10.1097/MIB.0000000000000452

[ajpa70005-bib-0061] Kuzawa, C. W. 2007. “Developmental Origins of Life History: Growth, Productivity, and Reproduction.” American Journal of Human Biology 19: 654–661. 17639581 10.1002/ajhb.20659

[ajpa70005-bib-0062] Larsen, C. S. 1997. Bioarchaeology. Interpreting Behaviour From the Human Skeleton. Cambridge: Cambridge University Press.

[ajpa70005-bib-0063] Lewis, M. 2018. “Fetal Paleopathology: An Impossible Discipline?” In The Anthropology of the Fetus: Biology, Culture, and Society, edited by S. Han , T. K. Betsinger , and A. B. Scott , 112–131. New York: Berghan Books.

[ajpa70005-bib-0064] Lockhart, P. B. , A. F. Bolger , P. N. Papapanou , et al. 2012. “Periodontal Disease and Atherosclerotic Vascular Disease: Does the Evidence Support an Independent Association? A Scientific Statement From the American Heart Association.” 125, no. 20: 2520–2544. 10.1161/CIR.0b013e31825719f3 22514251

[ajpa70005-bib-0065] Lopuhaa, C. E. , T. J. Roseboom , C. Osmond , et al. 2000. “Atopy, Lung Function and Obstructive Airways Disease in Adults After Prenatal Exposure to the Dutch Famine.” Thorax 55: 555–561. 10856314 10.1136/thorax.55.7.555 PMC1745806

[ajpa70005-bib-0066] Lu, A. , L. Petrullo , S. Carrera , J. Feder , I. Schneider‐Crease , and N. Snyder‐Mackler . 2019. “Developmental Responses to Early‐Life Adversity: Evolutionary and Mechanistic Perspectives.” Evolutionary Anthropology 28: 249–266. 31498945 10.1002/evan.21791

[ajpa70005-bib-0067] Mahoney Swales, D. 2019. “A Biocultural Analysis of Mortuary Practices in the Later Anglo‐Saxon to Anglo‐Norman Black Gate Cemetery, Newcastle‐Upon‐Tyne, England.” International Journal of Osteoarchaeology 29: 198–219.

[ajpa70005-bib-0068] McIntyre, L. , and G. Bruce . 2010. “Excavating all Saint's. A Medieval Church Rediscovered.” Current Archaeology 245: 30–37.

[ajpa70005-bib-0069] McKillup, S. 2012. Statistics Explained: An Introductory Guide for Life Scientists. Second ed. Cambridge: Cambridge University Press.

[ajpa70005-bib-0070] McPherson, C. B. 2021. “Examining Developmental Plasticity in the Skeletal System Through a Sensitive Developmental Windows Framework.” American Journal of Physical Anthropology 176, no. 2: 163–178. 34105143 10.1002/ajpa.24338

[ajpa70005-bib-0071] McPherson, C. B. , L. O'Donnell , E. Moes , and H. Edgar . 2024. “No Relationship Found Between Dental Fluctuating Asymmetry, Birthweight, and Birth Term in Two Modern North American Samples.” American Journal of Human Biology 36, no. 9: e24114. 10.1002/ajhb.24114. 38842218 PMC11623129

[ajpa70005-bib-0072] Metcalfe, N. B. , and P. Monaghan . 2001. “Compensation for a Bad Start: Grow Now, Pay Later?” Trends in Ecology & Evolution 16, no. 4: 254–260. 11301155 10.1016/s0169-5347(01)02124-3

[ajpa70005-bib-0073] Milner, G. R. , S. D. Ousley , J. l. Boldsen , S. M. Getz , S. Weise , and P. Tarp . 2019. *Transition Analysis 3 (TA3) Trait Manual. Public Distribution Draft (Ver. 1.0)*. National Institute of Justice.

[ajpa70005-bib-0074] Moes, E. , C. W. Kuzawa , and H. J. H. Edgar . 2024. “Sex‐Specific Effects of Environmental Temperature During Gestation on Fluctuating Asymmetry in Deciduous Teeth.” American Journal of Biological Anthropology 184: e24944. 38623790 10.1002/ajpa.24944

[ajpa70005-bib-0075] Neel, J. V. 1962. “Diabetes Mellitus: A Thrifty Genotype Rendered Detrimental by ‘Progress’?” American Journal of Human Genetics 14: 353–362. 13937884 PMC1932342

[ajpa70005-bib-0076] Newman, S. L. 2016. The Growth of a Nation: Child Health and Development in the Industrial Revolution in England, c. AD 1750–1850. Durham theses: Durham University.

[ajpa70005-bib-0078] Nolan, J. , B. Harbottle , and J. Vaughan . 2010. “The Early Medieval Cemetery at the Castle Newcastle Upon Tyne.” Archaeologia Aeliana 39: 147–287.

[ajpa70005-bib-0079] O'Donnell, L. , and E. Moes . 2020. “Increased Dental Fluctuating Asymmetry Is Associated With Active Skeletal Lesions, but Not Mortality Hazards in the Precontact Southwest United States.” American Journal of Physical Anthropology 175: 156–171. 33368176 10.1002/ajpa.24202

[ajpa70005-bib-0080] Ogden, A. 2008. “Advances in the Palaeopathology of the Teeth and Jaws.” In Advances in Human Palaeopathology, edited by R. Pinhasi and S. Mays , 283–307. Chichester: John Wiley.

[ajpa70005-bib-0081] Oishi, Y. , and I. Manabe . 2018. “Macrophages in Inflammation, Repair and Regeneration.” International Immunology 30, no. 11: 511–528. 30165385 10.1093/intimm/dxy054

[ajpa70005-bib-0082] Olsen, A. M. 2015. “Digitizing With StereoMorph How to Collect 2D Landmark and Curve Data From Photographs Using the StereoMorph Digitizing App.” *R Package Version 1.6.1* .

[ajpa70005-bib-0083] Olsen, A. M. , and M. W. Westneat . 2015. “StereoMorph: An R Package for the Collection of 3D Landmarks and Curves Using a Stereo Camera Set‐Up.” Methods in Ecology and Evolution 6: 351–356.

[ajpa70005-bib-0084] Palmer, A. R. 1994. “Fluctuating Asymmetry Analyses: A Primer.” In Developmental Instability: Its Origins and Evolutionary Implications, edited by T. A. Markow , 335–364. Dordrecht: Kluwer.

[ajpa70005-bib-0085] Palubeckaite, Z. , R. Jankauskas , Y. Ardagna , et al. 2006. “Dental Status of Napoleon's Great Army's (1812) Mass Burial of Soldiers in Vilnius: Childhood Peculiarities and Adult Dietary Habits.” International Journal of Osteoarchaeology 16: 355–365.

[ajpa70005-bib-0086] Pomeroy, E. , J. T. Stock , S. Stanojevic , J. J. Miranda , T. J. Cole , and J. C. K. Wells . 2012. “Trade‐Offs in Relative Limb Length Among Peruvian Children: Extending the Thrifty Phenotype Hypothesis to Limb Proportions.” PLoS One 7, no. 12: e51795. 23272169 10.1371/journal.pone.0051795 PMC3521697

[ajpa70005-bib-0087] Prince‐Buitenhuys, J. R. , and E. J. Bartelink . 2021. “Niche Construction Theory in Bioarchaeology.” In Theoretical Approaches in Bioarchaeology, edited by C. M. Cheverko , J. R. Prince‐Buitenhuys , and M. Hubbe , 93–112. Oxford: Routeledge.

[ajpa70005-bib-0088] Ravelli, A. C. J. , J. H. P. van der Meulen , R. P. J. Michels , et al. 1998. “Glucose Tolerance in Adults After Prenatal Exposure to the Dutch Famine.” Lancet 35: 173–177. 10.1016/s0140-6736(97)07244-9 9449872

[ajpa70005-bib-0089] Raynor, C. , R. McCarthy , and S. Clough . 2011. “Coronation Street, South Shields, Tyne and Wear. Archaeological Excavation and Osteological Analysis Report.” Lancaster: Oxford Archaeology North.

[ajpa70005-bib-0090] Reid, D. J. , and M. C. Dean . 2006. “Variation in Modern Human Enamel Formation Times.” Journal of Human Evolution 50: 329–346. 16300817 10.1016/j.jhevol.2005.09.003

[ajpa70005-bib-0091] Roberts, C. 2019. “Infectious Disease: Introduction, Periostosis, Periostitis, Osteomyelitus and Septic Arthritis.” In Ortner's Identification of Pathological Conditions in Human Skeletal Remains, edited by J. E. Buikstra , 285–320. London: Academic Press.

[ajpa70005-bib-0092] Roberts, C. A. , and J. E. Buikstra . 2019. “Bacterial Infections.” In Ortner's Identification of Pathological Conditions in Human Skeletal Remains, edited by J. E. Buikstra , 321–440. London: Academic Press.

[ajpa70005-bib-0093] Roseboom, T. J. , J. H. P. van der Meulen , C. Osmond , et al. 2000. “Coronary Heart Disease in Adults After Prenatal Exposure to the Dutch Famine.” Heart (British Cardiac Society) 84: 595–598. 11083734 10.1136/heart.84.6.595 PMC1729504

[ajpa70005-bib-0094] Roseboom, T. J. , J. H. P. van der Meulen , and A. C. J. Ravelli . 2001. “Effects of Prenatal Exposure to the Dutch Famine on Adult Disease in Later Life: An Overview.” Molecular and Cellular Endocrinology 185: 93–98. 11738798 10.1016/s0303-7207(01)00721-3

[ajpa70005-bib-0095] Scott, G. R. , and C. Turner . 1997. The Anthropology of Modern Human Teeth: Dental Morphology and Its Variation in Recent Human Populations. Cambridge: Cambridge University Press.

[ajpa70005-bib-0096] Selye, H. 1973. “The Evolution of the Stress Concept.” American Scientist 61: 692–699. 4746051

[ajpa70005-bib-0097] Slavin, P. 2013. “Market Failure During the Great Famine in England and Wales (1315‐1317).” Past and Present 222, no. 1: 9–49.

[ajpa70005-bib-0098] Stinson, S. 1985. “Sex Differences in Environmental Sensitivity During Growth and Development.” Yearbook of Physical Anthropology 28: 123–147.

[ajpa70005-bib-0099] Tan, L. Y. , T. V. Komarasamy , and V. R. M. T. Balasubramaniam . 2021. “Hyperinflammatory Immune Response and COVID‐19: A Double Edged Sword.” Frontiers of Immunology 12: 742941. 10.3389/fimmu.2021.742941 PMC8515020 34659238

[ajpa70005-bib-0100] Temple, D. H. 2014. “Plasticity and Constraint in Response to Early‐Life Stressors Among Late/Final Jomon Period Foragers From Japan: Evidence for Life History Trade‐Offs From Incremental Microstructures of Enamel.” American Journal of Physical Anthropology 155: 537–545. 25156299 10.1002/ajpa.22606

[ajpa70005-bib-0101] The Commissioners . 1845. “*Second Report of the Commissioners for Inquiring Into the State of Large Towns and Populous Districts, Appendix 2*.” London.

[ajpa70005-bib-0102] Tilliot, P. M. 1961. A History of the County of York: The City of York. Victoria History of the Counties of England. London: Oxford University Press.

[ajpa70005-bib-0103] Van Valen, L. 1961. “A Study of Fluctuating Asymmetry.” Evolution; International Journal of Organic Evolution 16: 125–142.

[ajpa70005-bib-0104] Vaupel, J. W. 1988. “Inherited Frailty and Longevity.” Demography 25, no. 2: 277–287. 3396752

[ajpa70005-bib-0105] Waldron, T. 1994. Counting the Dead. The Epidemiology of Skeletal Populations. London: Wiley.

[ajpa70005-bib-0106] Weisensee, K. E. 2013. “Assessing the Relationship Between Fluctuating Asymmetry and Cause of Death in Skeletal Remains: A Test of the Developmental Origins of Health and Disease Hypothesis.” American Journal of Human Biology 25: 411–417. 23559481 10.1002/ajhb.22390

[ajpa70005-bib-0107] Wells, J. C. K. 2007. “Flaws in the Theory of Adaptive Predictive Response.” Endocrinology and Metabolism 18, no. 9: 331–337. 10.1016/j.tem.2007.07.006 17951066

[ajpa70005-bib-0108] Wells, J. C. K. 2010. “Maternal Capital and the Metabolic Ghetto: An Evolutionary Perspective on the Transgenerational Basis of Health Inequalities.” American Journal of Human Biology 22: 1–17. 19844897 10.1002/ajhb.20994

[ajpa70005-bib-0109] Wells, J. C. K. 2012. “A Critical Appraisal of the Predictive Adaptive Response Hypothesis.” International Journal of Epidemiology 41: 229–235. 22422458 10.1093/ije/dyr239

[ajpa70005-bib-0110] Wells, J. C. K. 2016. The Metabolic Ghetto. An Evolutionary Perspective on Nutrition, Power Relations and Chronic Disease. Cambridge: Cambridge University Press.

[ajpa70005-bib-0111] Wells, J. C. K. , J. M. DeSilva , and J. T. Stock . 2012. “The Obstetric Dilemma: An Ancient Game of Russian Roulette, or a Variable Dilemma Sensitive to Ecology?” Yearbook of Physical Anthropology 55: 40–71. 10.1002/ajpa.22160 23138755

[ajpa70005-bib-0112] Weston, D. 2008. “Investigating the Specificity of Periosteal Reactions in Pathology Museum Specimens.” American Journal of Physical Anthropology 137, no. 1: 48–59. 18398845 10.1002/ajpa.20839

[ajpa70005-bib-0113] Weston, D. 2012. “Nonspecific Infection in Paleopathology: Interpreting Periosteal Reactions.” In A Companion to Paleopathology, edited by A. L. Grauer , 492–512. Chichester: Wiley‐Blackwell.

[ajpa70005-bib-0114] White, T. D. , and P. A. Folkens . 2005. The Human Bone Manual. San Diego: Elsevier.

[ajpa70005-bib-0115] Wigley, B. R. , E. C. Stillman , and E. Craig‐Atkins . 2024. “Taking Shape: A New Geometric Morphometric Approach to Quantifying Dental Fluctuating Asymmetry and Its Application to the Evaluation of Developmental Stress.” Archaeometry 66, no. 6: 1399–1423. 10.1111/arcm.12973.

[ajpa70005-bib-0116] Wood, J. W. , G. R. Milner , H. C. Harpending , and K. M. Weiss . 1992. “The Osteological Paradox: Problems of Inferring Prehistoric Health From Skeletal Samples.” Current Anthropology 33: 343–370.

[ajpa70005-bib-0117] Yaussy, S. 2024. “Using Craniofacial Fluctuating Asymmetry to Examine the Effects of Sex, Socioeconomic Status, and Early Life Experiences on Adult Age at Death in Industrial England.” American Journal of Biological Anthropology 184: e24907. 38380869 10.1002/ajpa.24907

[ajpa70005-bib-0118] Zietek, T. , and E. Rath . 2016. “Inflammation Meets Metabolic Disease: Gut Feeling Mediated by GLP‐1.” Frontiers in Immunology 7: 154. 27148273 10.3389/fimmu.2016.00154 PMC4840214

