# Supplementary material for: What Doesn't Kill You Makes You Stronger? Examining Relationships Between Early‐Life Stress, Later‐Life Inflammation and Mortality Risk in Skeletal Remains
Source: Am J Biol Anthropol. 2025 Feb 5;186(2):e70005. doi: 10.1002/ajpa.70005 (PMC11795231; doi:10.1002/ajpa.70005)
Supplement: Supplementary file 1 — Data S1 Supporting Information. [file AJPA-186-e70005-s001.docx]

**Supporting Information**

**AGE ESTIMATION**

**Comparisons between Sexes**

| Age categories | Female | Male | Combined |
| --- | --- | --- | --- |
| 15.0-20.0 | 3 (10.0%) | 1 (2.6%) | 4 (5.8%) |
| 20.1-25.0 | 2 (6.7%) | 10 (25.6%) | 12 (17.4%) |
| 25.1-30.0 | 2 (6.7%) | 3 (7.7%) | 5 (7.3%) |
| 30.1-35.0 | 0 (0.0%) | 3 (7.7%) | 3 (4.3%) |
| 35.1-40.0 | 4 (13.3%) | 2 (5.1%) | 6 (8.7%) |
| 40.1-45.0 | 2 (6.7%) | 6 (15.4%) | 8 (11.6%) |
| 45.1-50.0 | 4 (13.3%) | 3 (7.7%) | 7 (10.1%) |
| 50.1-55.0 | 3 (10.0%) | 6 (15.4%) | 9 (13.0%) |
| 55.1-60.0 | 7 (23.3%) | 3 (7.7%) | 10 (14.5%) |
| 60.1-65.0 | 1 (3.0%) | 1 (2.6%) | 2 (2.9%) |
| 65.1-70.0 | 0 (0.0%) | 0 (0.0%) | 0 (0.0%) |
| 70.1-75.0 | 1 (3.0%) | 1 (2.6%) | 2 (2.9%) |
| 75.1-80.0 | 0 (0.0%) | 0 (0.0%) | 0 (0.0%) |
| 80.1-85.0 | 0 (0.0%) | 0 (0.0%) | 0 (0.0%) |
| 85.1-90.0 | 0 (0.0%) | 0 (0.0%) | 0 (0.0%) |
| >90 | 1 (3.0%) | 0 (0.0%) | 1 (1.5%) |
| Total | 30 | 39 | 69 |

A tabular summary of mortality differences between females and males. Percentage in brackets.

**FA**

|  | No. | Min | Q1 | Median | Mean | Q3 | Max | Std dev |
| --- | --- | --- | --- | --- | --- | --- | --- | --- |
| M1 FA | 216 | -1.77 | -0.74 | -0.12 | 0.00 | 0.59 | 2.70 | 1.0 |

A summary of M1 FA scores.

**Comparisons between Sites**

| Site | No. | Min | Q1 | Median | Mean | Q3 | Max | Std dev |
| --- | --- | --- | --- | --- | --- | --- | --- | --- |
| BG | 84 | -1.49 | -0.69 | -0.17 | 0.06 | 0.67 | 2.73 | 0.98 |
| SS | 33 | -1.23 | -0.49 | -0.12 | 0.16 | 0.91 | 1.98 | 0.90 |
| WS | 29 | -1.72 | -0.49 | 0.28 | 0.361 | 0.90 | 2.01 | 1.07 |
| YB | 70 | -1.77 | -0.93 | -0.33 | -0.19 | 0.50 | 2.01 | 0.96 |

A comparison of M1 FA scores between sites.

**Comparisons between Sexes**

| Sex | No. | Min | Q1 | Median | Mean | Q3 | Max | Std dev |
| --- | --- | --- | --- | --- | --- | --- | --- | --- |
| female | 36 | -1.77 | -0.87 | -0.64 | -0.50 | -0.00 | 0.89 | 0.59 |
| male | 49 | -1.72 | -0.99 | -0.49 | -0.29 | 0.49 | 1.82 | 0.90 |

A comparison of M1 FA scores between sexes.

**Comparisons between Immature/Mature Skeletons**

| Maturity | No. | Min | Q1 | Median | Mean | Q3 | Max | Std dev |
| --- | --- | --- | --- | --- | --- | --- | --- | --- |
| immature | 104 | -1.72 | -0.47 | 0.15 | 0.36 | 1.11 | 2.73 | 1.04 |
| mature | 112 | -1.77 | -0.87 | -0.46 | -0.27 | 0.28 | 1.82 | 0.82 |

A comparison of M1 FA scores between immature and mature skeletons.

**PNBF**

Fifty-six (CPR=28.1%) skeletons and 157 bones (TPR=10.5%) exhibited plaques of PNBF. Of the individuals with PNBF, 38 (CPR=67.9%) had lesions with an active appearance, while 18 (CPR=32.1%) had remodelled lesions. In 46 cases it could be determined whether PNBF was distributed unilaterally or bilaterally; in 34 (CPR=73.9%) individuals, lesions were observed bilaterally.

**Comparisons between Sites**

| Site | PNBF remodelled (CPR) | PNBF active (CPR) | Total |
| --- | --- | --- | --- |
| BG | 6 (40.0%) | 9 (60.0%) | 15 |
| SS | 1 (14.3%) | 6 (85.7%) | 7 |
| WS | 1 (16.7%) | 5 (83.3%) | 6 |
| YB | 10 (35.7%) | 18 (64.3%) | 28 |

Frequency of skeletons with PNBF that had active lesions compared to remodelled by site.

| Site | PNBF unilateral (CPR) | PNBF bilateral (CPR) | Total |
| --- | --- | --- | --- |
| BG | 2 (14.3%) | 12 (85.7%) | 14 |
| SS | 1 (20.0%) | 4 (80.0%) | 5 |
| WS | 0 (0.0%) | 6 (100.0%) | 6 |
| YB | 9 (42.9%) | 12 (57.1%) | 21 |

Frequency of skeletons with PNBF that had unilateral and bilateral lesions by site.

**Comparisons between Sexes**

| Sex | PNBF absent (CPR) | PNBF present (CPR) | Total |
| --- | --- | --- | --- |
| female | 26 (74.3%) | 9 (25.7%) | 35 |
| male | 27 (60.0%) | 18 (40.0%) | 45 |

A tabular comparison of the frequency of skeletons with PNBF absent and present by sex.

| Sex | PNBF absent (TPR) | PNBF present (TPR) | Total |
| --- | --- | --- | --- |
| female | 267 (89.0%) | 33 (11.0%) | 300 |
| male | 351 (86.9%) | 53 (13.1%) | 404 |

A tabular comparison of the frequency of bones with PNBF absent and present by sex.

| Sex | PNBF remodelled (CPR) | PNBF active (CPR) | Total |
| --- | --- | --- | --- |
| female | 3 (34.4%) | 6 (66.6%) | 9 |
| male | 9 (50.0%) | 9 (50.0%) | 18 |

Frequency of skeletons with PNBF that had active lesions compared to remodelled by sex.

| Sex | PNBF unilateral (CPR) | PNBF bilateral (CPR) | Total |
| --- | --- | --- | --- |
| female | 2 (25.0%) | 6 (75.0%) | 8 |
| male | 5 (29.4%) | 12 (70.6%) | 17 |

A tabular comparison of the frequency of skeletons with PNBF that have unilateral and bilateral lesions by sex.

**Comparisons between Immature/Mature Skeletons**

| Maturity | PNBF absent (CPR) | PNBF present (CPR) | Total |
| --- | --- | --- | --- |
| immature | 72 (74.2%) | 25 (25.8%) | 97 |
| mature | 71 (69.6%) | 31 (30.4%) | 102 |

A tabular comparison of the frequency of skeletally mature and immature individuals with PNBF absent and present by life stage.

| Maturity | PNBF absent (TPR) | PNBF present (TPR) | Total |
| --- | --- | --- | --- |
| immature | 549 (89.4%) | 65 (10.6%) | 614 |
| mature | 783 (89.5%) | 92 (10.5%) | 875 |

A tabular comparison of the frequency of bones from skeletally mature and immature individuals with PNBF absent and present by life stage.

**PD**

Ninety-seven (CPR=44.9%) individuals exhibited some degree of alveolar degeneration and 377 (TPR=37.0%) tooth sockets were affected. Of the 97 skeletons with PD, 76 (CPR=78.3%) manifested the mildest markers of degeneration, 19 (CPR=19.6%) had moderate signs, and only two (CPR=2.1%) were affected by the most severe osteological indicators.

**Comparisons between Sexes**

| Sex | PD absent (CPR) | PD present (CPR) | Total |
| --- | --- | --- | --- |
| female | 8 (22.2%) | 28 (77.8%) | 36 |
| male | 10 (20.4%) | 39 (79.6%) | 49 |

A tabular comparison of the frequency of skeletons with PD absent and present by sex.

| Sex | PD absent (TPR) | PD present (TPR) | Total |
| --- | --- | --- | --- |
| female | 94 (40.5%) | 138 (59.5%) | 232 |
| male | 149 (50.0%) | 149 (50.0%) | 298 |

A tabular comparison of the frequency of teeth with surrounding alveolar bone with PD absent and present by sex.

| Site | PD mild (CPR) | PD moderate (CPR) | PD severe (CPR) | Total |
| --- | --- | --- | --- | --- |
| female | 22 (78.6%) | 6 (21.4%) | 0 (0.0%) | 28 |
| male | 28 (71.8%) | 9 (23.1%) | 2 (5.1%) | 39 |

Severity of lesions in females and males with PD.

**Immature/Mature Skeletons Compared**

| Maturity | PD mild (CPR) | PD moderate (CPR) | PD severe (CPR) | Total |
| --- | --- | --- | --- | --- |
| immature | 14 (93.3%) | 1 (6.7%) | 0 (0.0%) | 15 |
| mature | 62 (75.6%) | 18 (21.9%) | 2 (2.4%) | 82 |

A tabular comparison of lesion severity in skeletally mature and immature individuals with PD.
